# *De novo* Transcriptome Profiling of Flowers, Flower Pedicels and Pods of *Lupinus luteus* (Yellow Lupine) Reveals Complex Expression Changes during Organ Abscission

**DOI:** 10.3389/fpls.2017.00641

**Published:** 2017-05-02

**Authors:** Paulina Glazinska, Waldemar Wojciechowski, Milena Kulasek, Wojciech Glinkowski, Katarzyna Marciniak, Natalia Klajn, Jacek Kesy, Jan Kopcewicz

**Affiliations:** ^1^Department of Biology and Environmental Science, Nicolaus Copernicus UniversityTorun, Poland; ^2^Centre for Modern Interdisciplinary Technologies, Nicolaus Copernicus UniversityTorun, Poland

**Keywords:** yellow lupine, RNA-Seq, DEGs, flower, pod, abscission, microRNA

## Abstract

Yellow lupine (*Lupinus luteus* L., Taper c.), a member of the legume family (*Fabaceae* L.), has an enormous practical importance. Its excessive flower and pod abscission represents an economic drawback, as proper flower and seed formation and development is crucial for the plant's productivity. Generative organ detachment takes place at the basis of the pedicels, within a specialized group of cells collectively known as the abscission zone (AZ). During plant growth these cells become competent to respond to specific signals that trigger separation and lead to the abolition of cell wall adhesion. Little is known about the molecular network controlling the yellow lupine organ abscission. The aim of our study was to establish the divergences and similarities in transcriptional networks in the pods, flowers and flower pedicels abscised or maintained on the plant, and to identify genes playing key roles in generative organ abscission in yellow lupine. Based on *de novo* transcriptome assembly, we identified 166,473 unigenes representing 219,514 assembled unique transcripts from flowers, flower pedicels and pods undergoing abscission and from control organs. Comparison of the cDNA libraries from dropped and control organs helped in identifying 1,343, 2,933 and 1,491 differentially expressed genes (DEGs) in the flowers, flower pedicels and pods, respectively. In DEG analyses, we focused on genes involved in phytohormonal regulation, cell wall functioning and metabolic pathways. Our results indicate that auxin, ethylene and gibberellins are some of the main factors engaged in generative organ abscission. Identified 28 DEGs common for all library comparisons are involved in cell wall functioning, protein metabolism, water homeostasis and stress response. Interestingly, among the common DEGs we also found an miR169 precursor, which is the first evidence of micro RNA engaged in abscission. A KEGG pathway enrichment analysis revealed that the identified DEGs were predominantly involved in carbohydrate and amino acid metabolism, but some other pathways were also targeted. This study represents the first comprehensive transcriptome-based characterization of organ abscission in *L. luteus* and provides a valuable data source not only for understanding the abscission signaling pathway in yellow lupine, but also for further research aimed at improving crop yields.

## Introduction

Yellow lupine (*Lupinus luteus* L.), similarly to other members of the *Fabaceae* family (*Fabaceae* L.), has an enormous practical importance. Lupine seeds contain a high storage protein level, which is why it is used as feedstock for the production of high-protein animal feed. Its symbiosis with nitrogen-fixing bacteria which support its growth and development makes this plant a natural fertilizer enriching the soil with nitrogen (Prusiński, 2007). As flower and seed formation and development in crops is crucial for their productivity, flower and pod abscission becomes a factor that reduces benefits from growing lupines (Van Steveninck, 1958, 1959; Prusiński, 2007; Wilmowicz et al., 2016). On the other hand, a moderate abscission level is an agronomically desirable characteristic, since an excessive number of fruits is inversely proportional to their quality (Dokoozlian and Peacock, 2001). In order to be able to closely control the process, full knowledge of the molecular mechanisms behind generative organ development and the signaling pathways leading to organ abscission in particular plants is required.

Abscission is the process of shedding vegetative or reproductive organs by a plant in response to developmental, hormonal, and environmental cues. This process occurs at a special layer of cells called the abscission zone (AZ), and consists in cell separation enabled by hydrolytic enzymes. Plants can abscise buds, branches, petioles, leaves, flowers and fruits, while this process can be affected by environmental factors such as temperature, light quality, disease, water stress, and nutrition (Ascough et al., 2005; Estornell et al., 2013). The abscission of plant organs is associated with changes in the auxin gradient across the AZ, which is affected by ethylene (ET). It occurs when the auxin level below the AZ is higher than its concentration above that zone (Roberts et al., 2002; Meir et al., 2010). There are four key steps in abscission: (1) the establishment of the AZ, (2) the acquisition of the competence to respond to abscission signals, (3) the activation of organ abscission, and (4) the formation of a protective layer (Kim, 2014). It has been found that before and during peduncle abscission the expression of multiple regulatory genes changes (Kim et al., 2016), and that this variation affects a number of transcription factors associated with auxin and ethylene pathways (Sundaresan et al., 2016). However, it is not only auxin and ethylene that are involved in organ dropping. Recent studies on jasmonate signaling pathway mutants *coronatine insensitive1*(*coi1*) and *jasmonate-ZIM domain*(*jazz*) showed that these hormones, too, take part in regulating flower abscission in *Nicotiana attenuate* (Oh et al., 2013). In *A. thaliana*, several genes associated with the process of organ separation were identified, these being: *BLADE ON PETIOLE* (*BOP*) (McKim et al., 2008), *HAESA* (*HAE*) (Jinn et al., 2000), *HAESA*-*LIKE2* (*HSL2*) (Stenvik et al., 2008), *CAST AWAY* (*CST*), *NEVERSHED* (*NEV*), *EVERSHED* (*EVR*), *INFLORESCENCE DEFICIENT IN ABSCISSION* (*IDA*). The first of them is responsible for AZ formation, the second one for AZ functioning, and the last one for the final stage of organ separation. Late abscission stages are associated with the activity of many cell wall modifying enzymes, such as: polygalacturonases (PGs) (González-Carranza et al., 2002), xyloglucan endotransglucosylases/hydrolases (XTH) (Singh et al., 2011), β-1,4-glucanases/cellulases (EGL, CEL) (del Campillo and Bennett, 1996), and expansins (EXP) (Belfield et al., 2005; reviewed by Kim, 2014). Recently, *BOP* expression (Frankowski et al., 2015) and the influence of abscisic acid (ABA) on the ethylene (ET) biosynthesis pathway (Wilmowicz et al., 2016) in the abscission zone of yellow lupine's generative organs have been described, but more detailed information about the molecular mechanisms underlying the plant organ abscission is still needed.

Next-generation sequencing (NGS), for example by using the Illumina platform, has become an efficient and powerful approach for functional genomic research, especially when working with non-model plants (Unamba et al., 2015). Recently, RNA-Seq has been widely used for global gene expression profile analyses of plant response to a variety of biotic and abiotic stresses, such as salt (Zhou et al., 2016) or cold (Wang et al., 2015). It has also been used to study the abscission of soybean leaves (Kim et al., 2015, 2016), tomato flowers (Sundaresan et al., 2016) and apple fruits (Ferrero et al., 2015).

In this study, we explored complex expression changes during flower and pod abscission in *de novo* assembled transcriptome datasets generated from six different libraries from three organ types of *L. luteus* cv. Taper. We aimed to isolate the unique transcripts and unigenes from flowers, flower pedicels and pods that undergo abscission and those that do not, and to identify the differentially-regulated genes (DEGs) involved in abscission. The main objective of our research was to improve our understanding of the molecular mechanism of generative organ abscission in yellow lupine and to find the common genes (a potential target for crop improvement) regulating this process.

## Materials and methods

**Figure 1:**
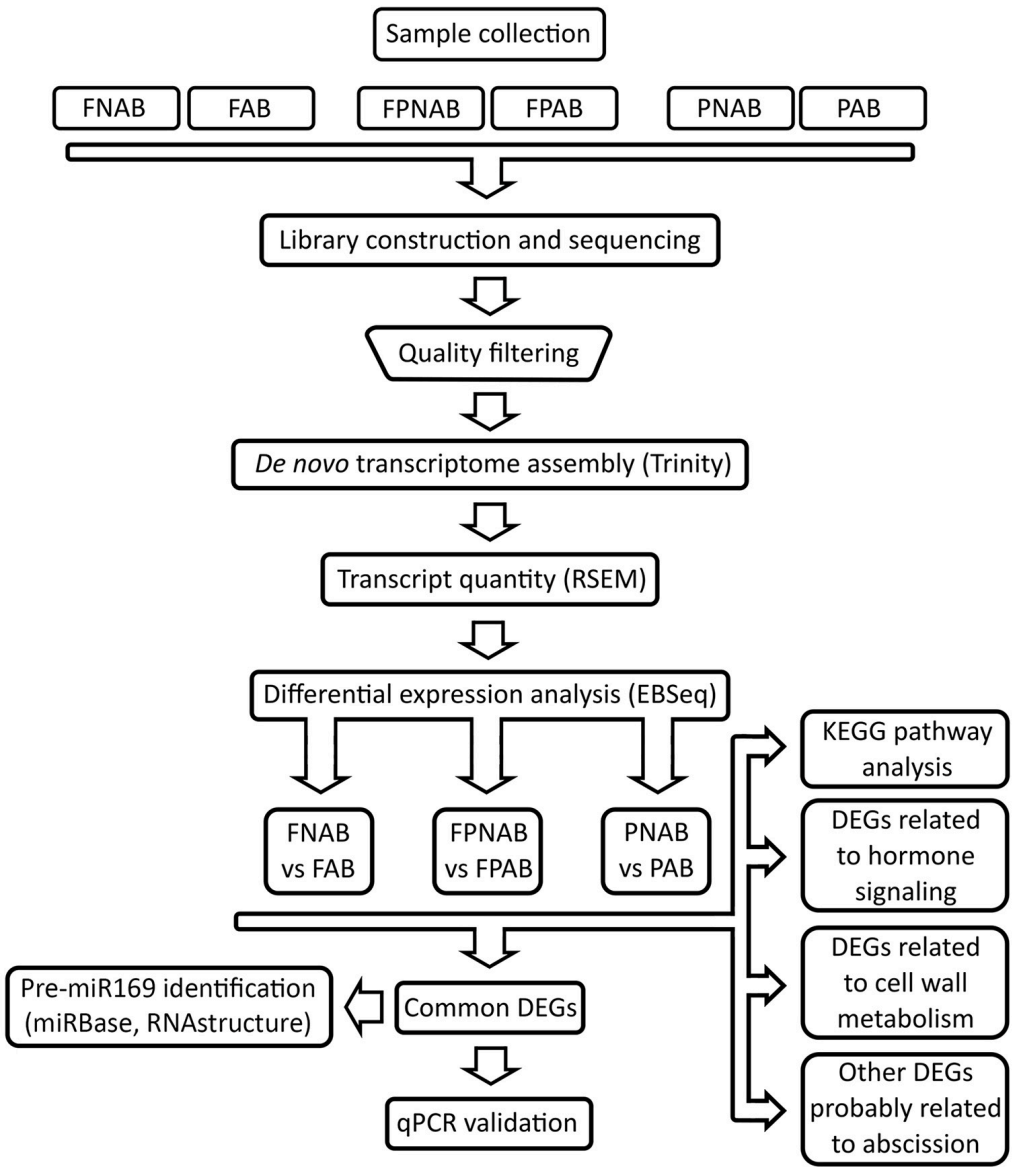
**Flowchart representing pipeline for RNA-seq experiments analysis conducted in the study**. FAB vs. FNAB-abscising flowers and non-abscising flowers, FPAB vs. FPNAB-abscising flower pedicels and non-abscising flower pedicels, PAB vs. PNAB-abscising pods and non-abscising pods.

Details of the experimental design and RNA Seq data analysis pipeline are summarized in Figure 1.

### Plant material

**Figure 2:**
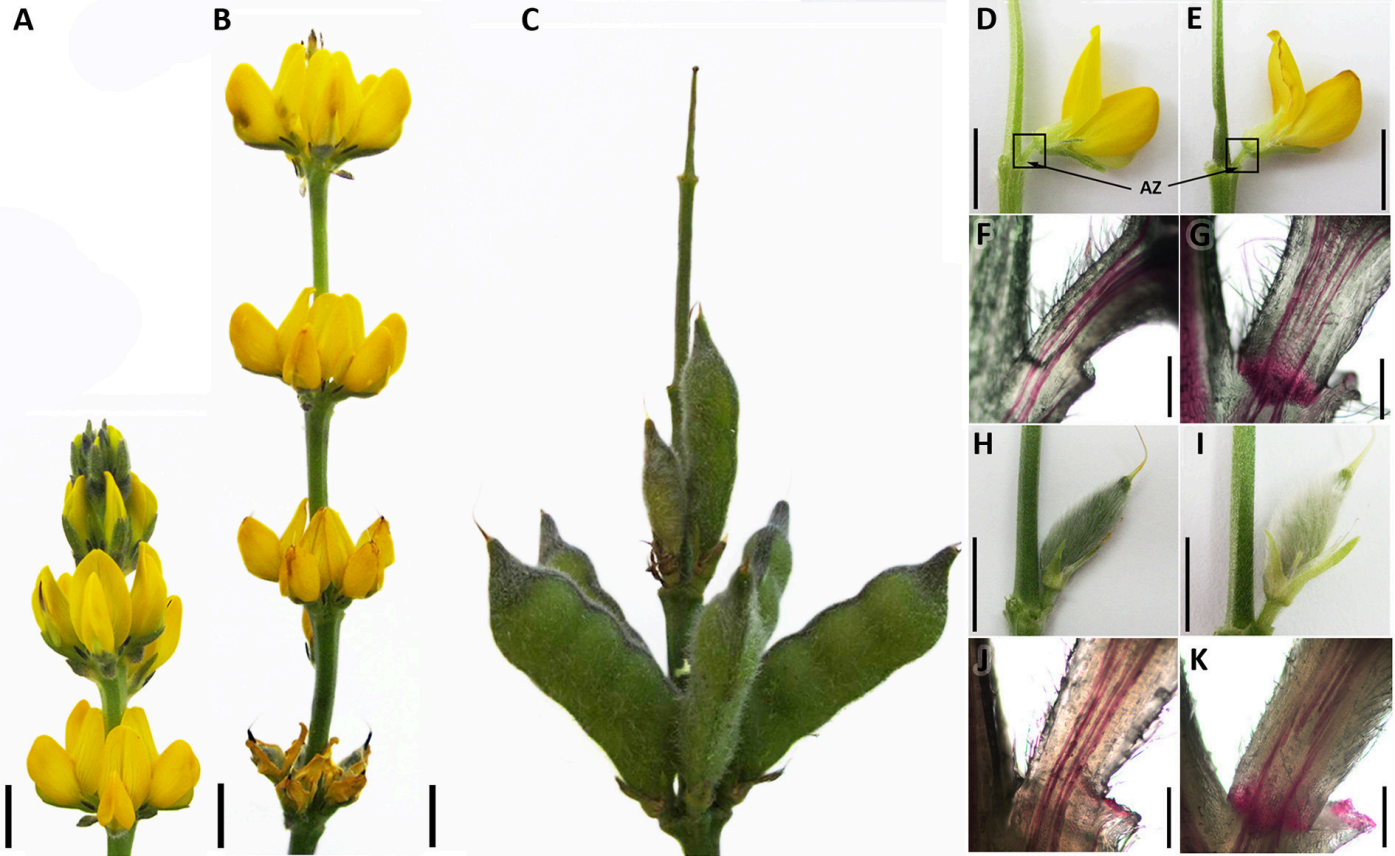
**Generative organs of *Lupinus luteus* cv. Taper**. Inflorescence development **(A–C)**. Developing and abscising flowers, **(D,E)**, respectively. Localization of harvested flower pedicels and abscission zones (AZ) indicated by squares and arrows, respectively. Cross-section view of developing and abscising flower pedicels after Phloroglucinol–HCl staining, **(F,G)**, respectively. View of developing and abscising pods, **(H,I)**, respectively. Cross-section view of developing and abscising pod pedicels after Phloroglucinol–HCl staining, **(J,K)**, respectively. *Black line* −1 cm bar for **(A–E**, **H,I)**, 600 nm bar for **(F,G,J,K)**.

For RNA-Seq analyses, different organs (flowers, pods and flower pedicels) from the yellow lupine (*L. luteus*) cultivar Taper cultivated in the growth chamber as previously described (Frankowski et al., 2015) were collected (Figure 2). From 54-day-old plants, whole fully opened flowers in stage 7 (Frankowski et al., 2015) with no signs of abscission (non-abscising flowers FNAB) were harvested (Figure 2D). Subsequently, their entire pedicels with an inactive abscission zone AZ located at the base of the flower pedicel (Figures 2D,F) were detached and collected as a separate sample (non-abscising flower pedicels FPNAB). From plants of the same age (54 days old), whole fully opened flowers with dehydrated petals (abscising flowers FAB) were harvested (Figure 2E). As above, their yellow and dehydrated pedicels with an active AZ (Figures 2E,G) were detached and collected as a separate sample (abscising flower pedicels FPAB). From 75 days-old plants pods with pedicels containing an inactive AZ (non-abscising pods PNAB) were harvested separately from the pods with an active AZ (abscising pods PAB) (Figures 2H–K). Following the harvest, the plant material was immediately frozen in liquid nitrogen and stored at −80°C until the RNA isolation procedures could be started.

To confirm localization and activation of AZ in harvested samples, we cut longitudinally five randomly taken flower and pod pedicels from each variant and stained them with phloroglucinol-HCl (Figures 2F,G,J,K) according to Tadeo and Primo-Millo (1990). We applied a saturated solution of phloroglucinol (Sigma-Aldrich) in 20% HCl directly to samples. Then images were taken using CX31 microscope (Olympus, Japan), SC50 camera and Olympus cellSens Entry 1.14 software (Olympus, Japan).

### cDNA library construction and illumina sequencing

Total RNA was isolated using the ISOLATE II RNA Plant Kit (Bioline, UK), following the manufacturer's protocol. The concentration and purity were measured with the ND-1000 spectrophotometer (NanoDrop, USA) before proceeding with further analyses. The integrity and quality of total RNA was evaluated using the Bioanalyzer 2100 (Agilent 2100 Technologies, USA) and agarose gel electrophoresis. Two independent isolations were performed from at least 5 plants (25 flowers, pedicels or pods) for each variant. For further procedures we used RNA samples with RNA integrity number (RIN) >8: two for both variants of pedicels (2 FPAB and 2 FPNAB) and one each for abscissive and nonabscissive flowers and pods (1 FAB, 1 FNAB, 1 PAB, 1 PNAB). Both the cDNA library construction, and the transcriptome sequencing performed on the HiSeq 2000 platform (Illumina Inc., USA), were carried out by Genomed (Poland). The sequence data generated in this study has been deposited at NCBI, the Sequence Read Archive (SRA) database under the accession number PRJNA285604 (BioProject), with the experiment accession number SRX1069734.

### Raw sequence processing and *De novo* assembly

The strategy used for data processing and differential gene expression analysis is presented in Figure 1. The preliminary sequence analysis, including adapter removal and low quality overhang deletion was, performed using Trimmomatic software (Fragkostefanakis et al., 2012). Trimmomatic settings were optimized for paired-end reads. After adapter removal and filtering out low quality reads, sequences shorter than 30 nt were discarded. The remaining sequences were analyzed using Trinity software (Grabherr et al., 2011) in accordance with the RNA-Seq analysis protocol published in Nature Protocols (Haas et al., 2013). At first, assembly based on *Lupinus angustifolius* (available at NCBI ID: 11024) (Yang et al., 2013) was considered, but due to the poor quality of its genome (191 thousands contigs) the idea was abandoned. *De novo* transcriptome assembly was performed using Trinity (Grabherr et al., 2011). The reference transcript assembly was obtained by joining individual reads into consensus sequences, which was then followed by the grouping of similar contigs into clusters using de Brujin graphs. Based on these graphs, genes and their isoforms (single or multiple) were obtained. Among the 219,514 reference sequences obtained (with a mean length of 418 bp), 166,477 genes were discovered. Each isoform has been given an id number consisting of the cluster number (c), the number of genes within the given cluster (g) and the isoform number (i). For example, c10_g3_i1 is the first isoform of the third gene in the tenth cluster. The final results were indexed in the R environment (R Development Core Team, 2008).

### Identification of differentially expressed genes

Reads from each separate sample were mapped as a collection of reference transcripts using the Bowtie aligner, and the quantity of each transcript in the sample was estimated using RSEM (part of the Trinity package) (Haas et al., 2013), using default settings for paired reads. The expression level was estimated at both unigene and isoform levels. Expression was described in three different ways: (1) the expected count: the number of reads per unigene, (2) TPM: the number of reads per unigene normalized to the library size (*Transcripts Per Million*), and (3) FPKM: the number of reads per unigene normalized to the library size and transcript length (*Fragments Per Kilobase Of Exon Per Million Fragments Mapped*).

Gene expression comparisons were performed using EBSeq software from the Bioconductor package (Leng and Kendziorski, 2015). This analysis was performed at the unigene level using the “expected count” expression type data. The following comparisons were made: FAB vs. FNAB, FPAB vs. FPNAB and PAB vs. PNAB. The obtained files were then filtered, leaving only those records where PPDE (Posterior Probability of Differential Expression) was less than 0.05 and Log_2_FC >2.

The sequence of each differentially expressed gene (DEG) was translated to the protein sequence, and then UniProt database (UniProt Consortium, 2015) was searched for sequences of similar proteins, and the best-matching protein was selected. If the protein ortholog could not be found, the unigene was assigned a “no hit” tag or left empty.

### Analysis of enriched KEGG pathways for DEGs

An analysis of overrepresented KEGG pathways (Kanehisa and Goto, 2000; Kanehisa et al., 2016) was performed using plantGSEA software, but due to the small number of statistically significant hits (data not shown) we decided to repeat the analysis using a different tool. Unfortunately, in the case of plants most of the popular tools cannot be used. We decided to perform the analysis manually, creating a series of Python scripts. The first step was to find proteins of a length of at least 30 amino-acids in the studied sequences, and for each gene only the longest protein was picked. The protein was not required to contain a start or stop codon, which was a reasonable assumption considering the fact that the investigated transcripts were much shorter (774 nt on average, compared to 1,557 nt in *A. thaliana* transcripts). Afterwards, we used the KAAS server (http://www.genome.jp/kaas-bin/kaas_main), which allowed us to assign orthologous groups from the KEGG database to the proteins and peptides that we had found. We used the following parameters: (i) the search method: BLAST, (ii) the species: *A*. *thaliana, G. max, V. vinifera, S. lycopersicum, O. sativa*, (iii) the method for finding potential homologs: SBH (single-directional best hit). Afterwards, we downloaded KEGG pathways for *Glycine max* (gmx) based on the KAAS server and on the obtained results, and performed an analysis of overrepresented KEGG pathways in the examined collection of genes (DEGs). For the purpose of this testing, the accurate Fisher test and FDR correction were used.

### Pre-miR169 identification

DEGs common for all of the compared transcriptomes that showed no homology to proteins were analyzed in respect of non-coding RNAs. The Basic Local Alignment Search Tool (BLAST) (Altschul et al., 1990) in the miRBase web database (ftp://mirbase.org/pub/mirbase/12.0/) was used to search for pre-miRNA and mature miRNA homologous sequences in these DEGs. The c125095_g1_i1 unigene has shown strong similarity to mature and stem-loop sequences of miR169 from various plant species. The unigene sequence was analyzed using Search miRBase Web software (Griffiths-Jones, 2004; Griffiths-Jones et al., 2006, 2008; Kozomara and Griffiths-Jones, 2011, 2014). RNAstructure software (http://rna.urmc.rochester.edu/RNAstructureWeb) was used to compute the secondary structure of the c125095_g1_i1 unigene sequence.

### Quantitative real-time RT-PCR analysis

A quantitative real-time RT- PCR (RT-qPCR) analysis was used to validate RNA-Seq results. For cDNA synthesis, 1 μg of total RNA was reverse-transcribed using the TranscriptMe Kit (DNA Gdansk, Poland). The synthesized cDNA samples were diluted 5 times prior to the qPCR. The qPCR was conducted using the KAPA Probe Fast Universal qPCR Kit (KAPA Biosystems, South Africa) following the manufacturer's protocol. Each reaction was prepared in a total volume of 20 μl consisting of 10 μl of 2 × KAPA Probe Fast qPCR Master Mix, 0.2 μM of each primer, 0.1 μM of a specific UPL probe and 5 μl of diluted cDNA. The real time qPCR was carried out on the LightCycler480 (Roche, Switzerland) under the following conditions: 95°C for 10 min, 45 cycles of 95°C for 10 s, 58°C for 30 s, and 72°C for 1 s. For each variant there were two biological and three technical replicates. *LlActin* was used as the reference gene. Primers and UPL (Universal Probe Library) probes used in the qPCR were designed using ProbeFinder version 2.48 (Roche, Switzerland) and are listed in Table S1. The data were analyzed using LightCycler480 software (Roche, Switzerland).

## Results

### RNA-seq and *De novo* transcriptome assembly

In order to obtain an insight into the process of generative organ abscission in yellow lupine we constructed transcriptome libraries: for flowers (FAB), flower pedicels (FPAB) and pods (PAB) undergoing abscission (with an active AZ), and the same for organs that did not fall (FNAB, FPNAB, PNAB). In order to verify whether AZ is active or inactive in chosen stage of flower and pod development, we analyzed their lignification pattern in pedicels by phloroglucinol staining—just before accomplishment of abscission, lignin accumulates within AZ (Clements and Atkins, 2001). In pedicels of not falling flowers and pods we observed no staining within AZ (only vascular bundles were stained), while organs undergoing abscission showed positive color reaction (Figures 2F,G,J,K). Because of similarities in flower and pod AZ structure, we decided to perform RNA-Seq analysis only for flower pedicels.

The HiSeq 2000 platform (Ilumina) generated from 56,784,288 to 46,619,042 raw reads in each library, which accounted for an average of 6.96 Gb raw data for each sample. About 98% of the raw reads were of high quality and, after Trimmomatic was run, about 80% of them were ultimately mapped (Table S2). The data were deposited in the National Center for Biotechnology Information's (NCBI) Short Read Archive (SRA) database under the accession number PRJNA285604.

**Figure 3:**
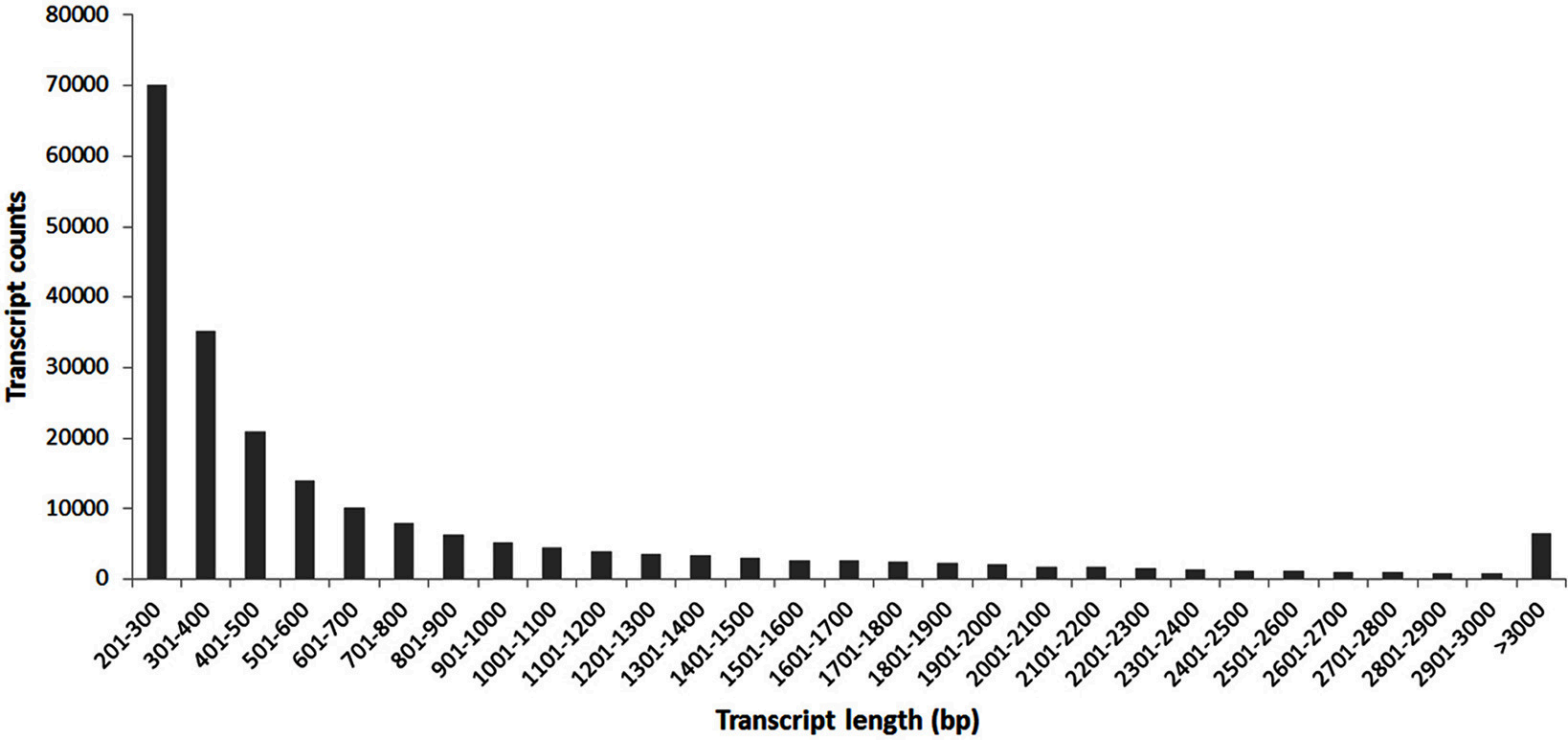
**The length distribution of the assembled transcripts**.

After *de novo* transcriptome assembly using Trinity software a set of 219,514 sequences of unique transcripts of a minimal length of 201 bp, including 166,473 different unigenes (the statistics of this process is summarized in Table 1), was obtained. Figure 3 shows the size distribution of the assembled transcripts, with the average size being 774 bp.

**Table 1:** **Statistics of the sequencing and assembly**.

**Items**	**Number**
Number of transcripts	219,514
Number of unigenes	166,473
Assembly length (Mb)	170
Minimum transcript length (bp)	201
Average transcript length (bp)	774
N50 (bp)	1,350

### Identification of DEGs in AB vs. NAB flowers, flower pedicels, and pods

In order to investigate molecular changes occurring during organ abscission in yellow lupine we compared transcriptomes of FAB vs. FNAB, FPAB vs. FPNAB PAB vs. PNAB transcriptomes. Literature data indicate, that hormonal changes and reorganization of cell wall structure play the most important role in organ abscission (Ascough et al., 2005; Corbacho et al., 2013; Estornell et al., 2013; Kim et al., 2015; Sundaresan et al., 2016) therefore in an analysis of the identified differentially expressed genes (DEGs) we focused on cell wall modification enzymes and hormone metabolism. We also took into account other DEGs that may be involved in organ fate. Finally, we identified DEGs that are common for all library comparisons.

**Figure 4:**
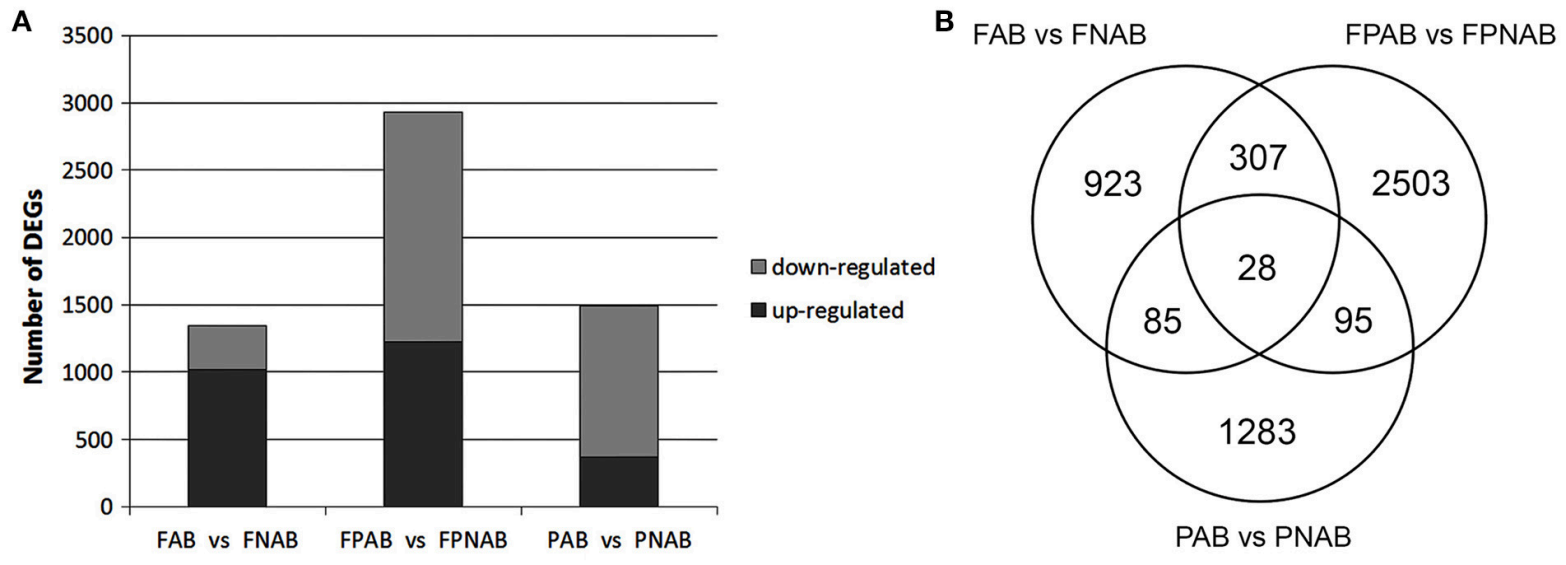
**Regulated characteristics of differentially expressed unigenes (DEGs) in each comparison**. **(A)** Three comparisons of *L*. *luteus* abscising flowers and non-abscising flowers (FAB vs. FNAB), abscising flower pedicels and non-abscising flower pedicels (FPAB vs. FPNAB), and abscising pods and non-abscising pods (PAB vs. PNAB) are shown. **(B)** Venn diagram showing the amounts of common and unique DEGs for the compared transcriptome libraries.

A bioinformatics analysis with EBseq of the transcriptomes revealed that during generative organ abscission in yellow lupine 5989 unigenes were either up- or down-regulated (PPDE < 0.05, FC > 2). The number of DEGs varied between library comparisons: 1343 in flowers (FAB vs. FNAB), 4.5% of which (293) were strongly affected (log_2_ ratio over 5); 2933 in flower pedicels (FPAB vs. FPNAB), 9.5% of which (308) were strongly affected; 1491 in pods (PAB vs. PNAB), 11.6% of which (128) were strongly affected (Figure 4A and Table S3). In abscising flowers, more unigenes were up-regulated (1018) than down-regulated (325), while in the flower pedicels and in the pods the trend was opposite: more unigenes were down-regulated (1,712 and 1,126, respectively) than up-regulated (1,221 and 365, respectively) (Figure 4A). The number of unique DEGs was the highest in the flower pedicels (2,503), being two times lower in the pods (1,283) and the lowest in the flowers (923). Flowers and pedicels shared 307, pedicels and pods 95, and pods and flowers 85 DEGs. Only 28 DEGs were common for all library comparisons (Figure 4B).

### DEGs involved in cell wall metabolism in abscising and maintained generative organs of lupine

In abscising flowers, flower pedicels and pods, genes encoding in cell wall hydrolyzing enzymes were mostly up-regulated.

In abscising flowers, 27 DEGs were involved in cell wall metabolism. These unigenes showed similarity to *EXPANSINS EXP* (3 DEGs), *XYLOGLUCAN ENDOTRANSGLUCOSYLASE / HYDROLASES XTH* (1), *ENDOGLUCANASES / CELLULASES EGL/CELL* (2), *POLYGALACTURONASES PG* (5), *BETA-GALACTOSIDASES BGal* (4), *PECTINESTERASES PME* (10), *PECTIN ACETYLESTERASES PAE* (1), and *PECTATE LYASES PEC* (1). Most of them (22 DEGs) were up-regulated, and only 5 down-regulated in FNAB (Table 2 and Table S4).

**Table 2:** **Differential expression patterns of cell wall-related genes in comparison of libraries FAB vs. FNAB, FPAB vs. FPNAB, and PAB vs. PNAB**.

**Gene description**	**FAB vs. FNAB regulated**		**FPAB vs. FPNAB regulated**		**PAB vs. PNAB regulated**	
	**Up**	**Down**	**Up**	**Down**	**Up**	**Down**
Expansin EXP	3	–	9	2	1	4
Xyloglucan endotransglucosylase/hydrolase XTH	1	–	9	9	1	4
Endoglucanase/Cellulase EGL/CELL	1	1	4	3	2	1
Polygalacturonase PG	5	–	18	7	–	10
Beta-galactosidase BGal	3	1	12	8	–	2
Pectinesterase PME	7	3	19	1	3	38
Pectin acetylesterase PAE	1	–	2	2	–	2
Pectate lyase PEC	1	–	8	–	–	8

Numerous DEGs (113) were involved in cell wall metabolism in the flower pedicels. The identified unigenes showed similarity to *EXP* (11 DEGs), *XTH* (18), *EGL*/*CELL* (7), PG (25), *BGal* (20), *PME* (20), *PAE* (4), and *PEC* (8). Most of them (81) were up-regulated, and only 32 down-regulated in FPNAB (Table 2 and Table S4).

In abscising pods, 76 DEGs were involved in cell wall metabolism. The identified unigenes showed similarity to *EXP* (5 DEGs), *XTH* (5 DEGs), *EGL* (3 DEGs), *PG* (10 DEGs), *BGal* (2 DEGs), *PME* (41 DEGs), *PAE* (2 DEGs), and *PEC* (8 DEGs). Most of them (69 DEGs) were down-regulated, and only 7 up-regulated in PNAB (Table 2 and Table S4).

### Differential regulation of genes involved in hormone signaling in compared generative organs

We observed differences in expression of genes involved in distribution and sensitivity to phytohormones during abscission of generative organs.

#### Auxin

Plants adopt different strategies to change the distribution pattern of active form of auxin across the tissues. Our study on yellow lupine revealed, that in generative organs undergoing abscission, following molecular mechanisms take place: (i) decrease in expression of auxin biosynthesis enzymes (like in FAB), (ii) and/or decrease in expression of its catabolism enzymes (like in FAB, PFAB), (iii) decrease in expression of enzymes that release auxin from its conjugates (like in PAB), (iv) increase (like in FPAB) and/or decrease in (like in FAB, FPAB) expression of its transport proteins. This resulted in complex changes in expression of genes involved in auxin signal transduction in all studied tissues.

In abscising flowers, 11 DEGs were involved in auxin (IAA) signaling (Table 3 and Table S5). Two enzymes up-regulated in FNAB ensure the right level of the active form of this phytohormone: YUCCA8 (c61021_g1), a key enzyme in IAA biosynthesis (Won et al., 2011), and 2-oxoglutarate-dependent dioxygenase (DAO) (c134360_g1), which is essential for auxin catabolism and the maintenance of auxin homeostasis in reproductive organs (Zhao et al., 2013). Only one unigene in this library comparison was responsible for auxin transport, *TORNADO 2* (c39845_g1) (Cnops, 2006), and it was up-regulated in FNAB. There were 8 DEGs involved in auxin signal transduction, three of which were down-regulated. Two DEGs displayed similarity to auxin response factor *ARF4* (c62886_g4) and *ARF7* (c61288_g4), and were up- and down-regulated, respectively.

**Table 3:** **Differential expression patterns of plant hormone metabolism and signaling-related genes in comparison of libraries FAB vs. FNAB**.

**Hormone**	**Total no. of DEGs**	**Up-regulated**	**Down-regulated**	**Function**
IAA	11	1	–	Biosynthesis
		1	–	Catabolism
		1	–	Transport
		5	3	Signal transduction
GA	8	3	–	Biosynthesis
		–	2	Catabolism
		3	–	Signal transduction
ET	3	2	1	Signal transduction
ABA	2	–	2	Signal transduction
JA	3	2	–	Biosynthesis
		–	1	Signal transduction
CK	1	1	–	Catabolism
BR	2	2	–	Signal transduction
SA	1	1	–	Signal transduction
SL	1	1	–	Signal transduction

Among DEGs identified in all library comparisons, most of the unigenes associated with auxin signaling (44) were differently expressed in the pedicels of flowers (Table 4 and Table S6). In the datasets for pedicels of the falling and the control flowers we found two DEGs responsible for auxin break-down: DAO (c134360_g1), essential for IAA oxidation, and indole-3-acetate O-methyltransferase 1 (c33445_g1), involved in methylation of the free carboxyl end of IAA. Both of them were over-expressed in FPNAB. Three of the four DEGs encoding transport proteins, namely auxin transporter-like protein LAX4 (c61059_g4) and LAX5 (c61634_g1, c11165_g1), were up-regulated in FPNAB. In this dataset we found two small auxin upregulated RNAs (SAURs), namely an up-regulated AGR7 (c50003_g2) and a down-regulated AGR2 (c59673_g5) in FPNAB. The list of DEGs identified in the pedicels was rich in genes encoding elements of auxin signal transduction. Two of the DEGs encoded receptors, namely a down-regulated F-box protein SKP2A (c54931_g4) and an up-regulated auxin-binding protein ABP19a (c58059_g1, c110842_g1, c52095_g1), three other DEGs encoded down-regulated ARF4 (c62886_g2), ARF7 (c128482_g1, c30002_g1) and ARF19 (c60211_g6), while as many as 15 DEGs encoded up- or down-regulated Auxin/Indole-3-Acetic Acid (AUX/IAA) proteins.

**Table 4:** **Differential expression patterns of plant hormone metabolism and signaling-related genes in comparison of libraries FPAB vs. FPNAB**.

**Hormone**	**Total no. of DEGs**	**Up-regulated**	**Down-regulated**	**Function**
IAA	44	2	–	Catabolism
		4	1	Transport
		22	15	Signal transduction
GA	12	3	1	Biosynthesis
		–	4	Catabolism
		4	–	Signal transduction
ET	17	–	3	Biosynthesis
		8	6	Signal transduction
ABA	9	1	–	Catabolism
		–	8	Signal transduction
JA	3	–	3	Biosynthesis
CK	9	2	–	Biosynthesis
		3	3	Catabolism
		1	–	Signal transduction
BR	8	3	2	Biosynthesis
		3	–	Signal transduction
SA	6	2	4	Signal transduction

Among the genes differentially expressed in the abscising and non-abscising pods (Table 5 and Table S7) we found one that encoded IAA-amino acid hydrolase ILL2 (c28519_g1) that catalyzes the hydrolysis of IAA conjugates and releases the active form of this hormone (LeClere et al., 2002) and was up-regulated in PNAB. Genes encoding auxin receptors TRANSPORT INHIBITOR RESPONSE 1 (TIR1) (c85345_g1), F-box protein SKP2A (c54931_g4) and auxin-binding protein ABP19a (c52095_g1, c58059_g1) were down-regulated. Simultaneously, two SAURs were down-regulated and one AUX/IAA was up-regulated in this dataset. Interestingly, two ARFs (5 DEGs), i.e., *ARF2* (c52420_g3, c52420_g4, c52420_g1) and *ARF4* (c62886_g7, c62886_g2), were significantly up-regulated in PNAB.

**Table 5:** **Differential expression patterns of plant hormone metabolism and signaling-related genes in comparison of libraries PAB vs. PNAB**.

**Hormone**	**Total no. of DEGs**	**Up-regulated**	**Down-regulated**	**Function**
IAA	14	1	–	Catabolism
		6	7	Signal transduction
GA	12	9	–	Biosynthesis
		1	–	Catabolism
		–	2	Signal transduction
ET	5	–	3	Biosynthesis
		–	2	Signal transduction
ABA	2	1	–	Catabolism
		1	–	Signal transduction
JA	6	4	–	Biosynthesis
		1	1	Signal transduction
SA	6	1	5	Signal transduction
SL	1	1	–	Signal transduction

#### Ethylene

We observed differences in expression of ethylene (ET) biosynthesis genes in flower pedicels and pods and of genes involved in ethylene signal transduction in all studied organs.

In flowers we found 3 DEGs involved in ET signal transduction, among them up-regulated Mitogen-activated protein kinase 6 (c65488_g1), earlier proven to be involved in leaf senescence (Zhou et al., 2009) (Table 3 and Table S5).

In the flower pedicels, 17 of the identified DEGs were involved in ET metabolism. Three unigenes encoding ET biosynthesis enzyme 1-aminocyclopropane-1-carboxylate oxidase (ACO) 1 (c60801_g4, c60409_g3, c140425_g1) were down-regulated in FPNAB. Eight of the fourteen genes encoding elements of ET signal transduction were up-regulated (Table 4 and Table S6).

In pods all DEGs attributed to ethylene biosynthesis encoding 1-aminocyclopropane-1-carboxylate synthase 1 (ACS1) (c60313_g3, c58986_g1) and ACS7 (c107301_g1) and to signal transduction encoding ethylene-responsive transcription factor 3 (ERF3) (c18833_g1) and ERF1A (c81001_g1) were less intensively expressed in PNAB (Table 5 and Table S7).

#### Gibberellin

We observed differences in expression of genes involved in gibberellin (GA) (i) biosynthesis: they were down-regulated in FAB and PAB, and mostly down-regulated in PFAB, except for the enzyme that catalyzes the transformation of direct precursor to GA active form (ii) catabolism: they were up-regulated in FAB and FPAB, and down-regulated in PAB (iii) signal transduction: they were down-regulated in FAB and up-regulated in PAB, and (iv) genes controlled by this hormone (down-regulated in FPAB).

As regards gibberelin eight DEGs in flowers were involved in GA signaling (Table 3 and Table S5). Two of them encode gibberellin 2-beta-dioxygenase 1 (GA2OX1) (c57884_g1, c25173_g1), which is engaged in GA catabolism and were down-regulated in FNAB. The rest of them were up-regulated and included genes encoding enzymes responsible for GA biosynthesis: ent-kaurenoic acid oxidase 2 (KAO2) (c78744_g1), gibberellin 20 oxidase 2 (GA20OX2) (c54588_g2), GA20OX1 (c54588_g1); and for signal transduction: GAI (c49118_g1), gibberellin-regulated protein 12 (c59846_g1), and 14 (c48197_g2).

In flower pedices we found four DEGs engaged in GA biosynthesis (Table 4 and Table S6). Three of them, namely *GA20OX2* (c54588_g2), *GA20OX1* (c54588_g1), and *KAO2* (c60896_g9), were up-regulated, while the remaining one, *LE* (c24709_g1), was down-regulated in FPNAB. Three DEGs encoding the GA2OX1 enzyme (c25173_g1, c57884_g1, c49763_g3) responsible for hydroxylation of the active form of GA (Martin et al., 1999), were down-regulated. Also, four DEGs similar to gibberellin-regulated proteins were up-regulated in FPNAB.

All 9 DEGs attributed to GA biosynthesis were much more intensively expressed in PNAB (Table 5 and Table S7). For example, the c59646_g2 unigene, *GA20OX2* with log_2_FC = 12.88, was the most up-regulated DEG in PNAB. Only one DEG involved in GA catabolism encoding GA2OX1 (c49763_g2) was up-regulated on a moderate level. DEGs involved in GA response encoding GA signal transduction transcription factor GAMYB (c40131_g1, c60985_g11) were significantly down-regulated in PNAB.

#### Abscisic acid

We observed differences in expression of genes involved in abscisic acid (ABA) catabolism (they were down-regulated in FPAB and PAB), and signal transduction (they were up-regulated in FAB and FPAB).

Two DEGs down-regulated in FNAB were involved in ABA signaling (Table 3 and Table S5). That DEGs indicate similarity to *PYL2* (c35977_g2 and c35977_g3) that are responsible for ABA perception (Yin et al., 2009).

Among the DEGs found in the flower pedicels and recognized as involved in ABA signaling (Table 4 and Table S6), *PYL1* (2 DEGs), *SAPK2* (1 DEG), *SAPK8* (1 DEG) and *PP2CA* (4 DEGs) were down-regulated in FPNAB. Simultaneously, abscisic acid 8'-hydroxylase 4 *CYP707A4* (c61127_g1) engaged in ABA break-down was up-regulated.

Additionally, ABA catabolism-related gene encoding abscisic acid 8′-hydroxylase 1 (CYP707A1) (c40794_g1) that catalyzes the first step of ABA degradation was up-regulated in PNAB (Table 5 and Table S7).

#### Jasmonate

We observed differences in expression of genes involved in jasmonate (JA) biosynthesis (they were down-regulated in FAB) and signal transduction (they were up-regulated in FAB, FPAB and down-regulated in PAB).

We found three DEGs associated with JA metabolism in flowers. Two of them were up-regulated in FNAB, and these were chloroplastic *LIPOXYGENASE 6* (*LOX6*) (c92415_g1) and *JASMONATE O-METHYLTRANSFERASE* (c121732_g1) that were involved in jasmonic acid (JA) and ester methyl jasmonate (JaMe) biosynthesis, respectively. The other one was more frequently expressed in FAB, namely *TIFY10A* (c52719_g1) engaged in JA signal transduction (Table 3 and Table S5).

Three DEGs encoding chloroplastic JA biosynthesis enzymes, namely LINOLEATE 9S-LIPOXYGENASE 5, LOX2 (c45959_g2, c57820_g1) and allene oxide synthase CYP74A (c92570_g1) were down-regulated in FPNAB (Table 4 and Table S6).

In contrast, DEGs associated with JA biosynthesis were all up-regulated in PNAB. The DEGs associated with JA biosynthesis, i.e., *JASMONATE O-METHYLTRANSFERASE* (c43486_g2), *LOX1* (c92206_g1, c70262_g1) and *LOX4* (c61214_g3), were up-regulated in PNAB (Table 5 and Table S7).

#### Brassinosteroid

Brassinosteroid (BR) signaling decreased in abscising flowers and their pedicels. In flower pedicels there were complex differences in expression of BR biosynthesis enzymes.

Two genes involved in BR signal transduction and one engaged in response to this hormone were significantly down-regulated in flowers (Table 3 and Table S5).

Among the 8 DEGs in the flower pedicels recognized as involved in brasinosteroid (BR) signaling, all the three involved in signal transduction were up-regulated in FPNAB (Table 4 and Table S6). The expression profile of DEGs putatively encoding BR biosynthesis enzymes was more complex: some of them were up- and others were down-regulated. Unigenes encoding the same protein, cytochrome P450 85A (CYP85A), and belonging to the same cluster were differentially expressed: c54957_g3 and c54957_g2 were up-regulated, while c54957_g1 was down-regulated in FPNAB. Other DEGs putatively encoding BR biosynthesis enzymes, namely 3-epi-6-deoxocathasterone 23-monooxygenase CYP90D1 (c51134_g1) and CYP85A1 (c57767_g3), were up- and down-regulated in FPNAB, respectively.

In pods there was not find any unigene related to BR metabolism.

#### Cytokinin

We observed differences in expression of genes involved in cytokinin (CK) (i) biosynthesis: they were down-regulated in FPAB, (ii) catabolism: they were down-regulated in FAB and either down- and up-regulated in FPAB, and (iii) signal transduction: they were down-regulated in FPAB.

In FAB vs. FNAB transcriptome comparison we found one DEG related to CK metabolism the gene encoding cytokinin hydroxylase CYP735A1 (c59901_g1) involved in CK catabolism (Table 3 and Table S5).

In flower pedicels (Table 4 and Table S6), among the 9 DEGs related to cytokinin (CK), two genes encoding enzymes that catalyze the final steps of CK biosynthesis, cytokinin riboside 5′-monophosphate phosphoribohydrolases LOG1 (c62014_g7) and LOG3 (c44862_g1) (Kuroha et al., 2009), were significantly up-regulated in FPNAB. Also, genes encoding cytokinin dehydrogenases that catalyze CK oxidation to a biologically inactive form displayed differential expression: two of them, *CKX5* (c62634_g1) and *CKX3* (c55747_g3, c55747_g1), were up-regulated, while the other two, namely *CKX1* (c50912_g1, c50912_g4) and *CKX9* (c108053_g1), were down-regulated in FPNAB. We found only one DEG assigned to cytokinin signal transduction representing the CK receptor, namely histidine kinase *AHK4* (c49543_g4), and it was up-regulated in FPNAB.

In pods there was not find any unigene related to CK metabolism.

#### Salicylic acid

Expression of genes involved in salicylic acid (SA) signal transduction was down-regulated in abscising flowers, and in other studied organs these differences were more complex.

One gene participating in salicylic acid signal transduction, namely pathogenesis-related protein PR-1 (c16844_g1), was up-regulated in FNAB (Table 3 and Table S5).

Among the DEGs identified in the flower pedicels as involved in salicylic acid signaling we found five unigenes (encoding 4 proteins) showing similarity to elements of signal transduction: one up-regulated pathogenesis-related protein PR1 (c16844_g1) and three down-regulated ones in FPNAB: transcription factor TGA1 (c8716_g1 and c53737_g2), Salicylic Acid-Binding Protein 2 (c93153_g1), and PRB1 (c33973_g2) (Table 4 and Table S6).

One of the six DEGs attributed to SA-related genes and belonging to PR1 gene family were over-expressed in PNAB, while the remaining five were down-regulated (Table 5 and Table S7).

#### Stigolactone

In flowers and pods transcriptome comparisons we found one DEG associated with strigolactone signal transduction (up-regulated in FNAB and PNAB), strigolactone esterase DAD2 (c47746_g1) that binds and hydrolyzes mobile strigolactones initiating SCF-mediated strigolactone signal transduction (Hamiaux et al., 2012) (Tables 3, 5, Tables S5, S7).

### Characterization of other DEGs probably related to generative organ abscission

#### Characterization of DEGs from abscised flowers and control

Among DEGs in flowers we also found genes encoding proteins directly involved in protection against reactive oxygen species (ROS) (Table S8), namely peroxidase 12 (c95461_g1, log_2_FC = 4.2) and peroxidase 10 (c108985_g1, log_2_FC = −4.6), as well as enzymes belonging to the carotenoid biosynthesis pathway, namely chloroplastic carotenoid cleavage dioxygenase 4 (c63538_g3, log_2_FC = 4.9), chloroplastic or chromoplastic zeta-carotene desaturase (c23470_g1, log_2_FC = 4.5) and chloroplastic beta-carotene hydroxylase 2 (c47712_g2, log_2_FC = 3.9).

The flowers that are dropped and those that are maintained on the plant also differ in respect of the genes involved in proper fertilization (Table S8). Some DEGs, such as NUCLEAR FUSION DEFECTIVE 4 (NDF4) (c41239_g1) encoding a protein required for fusion of polar nuclei during female gametophyte development and karyogamy during fertilization (Portereiko et al., 2006), or POLLENLESS3 (c47977_g1) which is essential for male fertility, especially for microspore and pollen grain production (Glover et al., 1998), were less abundantly expressed in FAB.

#### Characterization of other DEGs from flower pedicels with active AZ and control

As it was in the case of flowers, we found DEGs encoding elements engaged in protection against ROS, and these were 18 different peroxidases (10 up- and 8 down-regulated) and chloroplastic beta-carotene 3-hydroxylase (c57645_g2, log_2_FC = −2.7) (Table S9).

Additionally, one of the most intensively expressed DEGs in the flower pedicels with an active AZ showed similarity to vacuolar processing enzyme VPE (8 unigenes, log_2_FC from −2.69 to −7.09), an executor of plant PCD (Programmed Cell Death) (Hatsugai et al., 2004) (Table S9). VPE is a cysteine protease that cleaves a peptide bond at the C-terminal side of asparagine and aspartic acid (Wang et al., 2009) and plays an essential role in the regulation of the lytic system of plants during their defensive and developmental processes (Hara-Nishimura et al., 2005).

In the flower pedicels, we identified several transcription factors specific for soybean leaf and flower abscission (Kim et al., 2016), such as NAC (29 DEGs), WRKY (12 DEGs) and AIL/PLETHORA (PLT) (3 and 2 DEGs respectively) which were up-regulated in FPAB (Table S9).

Interestingly, transcripts of 12 DEGs encoding aquaporins belonging to tonoplast intrinsic protein (TIP) and plasma membrane intrinsic protein (PIP) gene families were more accumulated in the pedicels of non-abscising flowers (Table S9).

#### Characterization of other DEGs from abscising and maintained pods of lupine

Similarly to the case of flowers and flower pedicels, we found DEGs encoding 8 different peroxidases, 3 up- and 5 down-regulated, but no enzyme belonging to the carotenoid biosynthesis pathway (Table S10).

Other DEGs with a known homology, apart from the one that was the most up-regulated in the pods (gibberellin 20 oxidase), were those that showed similarity to genes encoding arabinogalactan proteins, i.e., PISTIL-SPECIFIC EXTENSIN-LIKE protein (c33p206_g1, log_2_FC = 9.5) and FASCICLIN-LIKE ARABINOGALACTAN PROTEIN 10 (FLA) (c141625_g1, log_2_FC = 8.8) (Table S10). In tomato these types of protein are preferentially expressed in immature white fruits (Fragkostefanakis et al., 2012).

During data interpretation special attention was paid to the AGL62 Agamous-like MADS-box protein (c59718_g1, log_2_FC = 8.6) (Table S10). The AGL62 suppresses cellularization during endosperm development in *Arabidopsis* (Kang et al., 2008).

Another DEG that could play a role in pod abscission or development is transcription factor MYC4 (c96574_g1, log_2_FC = 5,25; Table S10). Recent studies showed that MYC4 together with MYC2 and MYC3 are involved in regulating seed production in a JA-dependent manner (Qi et al., 2015).

By analyzing the same dataset we discovered, that in the pods undergoing abscission (PAB) as many as 26 unigenes related to calcium transport, homeostasis and response were over-expressed (log_2_FC varied from −2.86 to −4.68). This set of genes encompassed putative calcium-transporting ATPase (1 DEG), calcium-dependent protein kinases (6 DEGs), probable calcium-binding proteins (12 DEGs), cation/calcium exchanger (1 DEG) and calmodulin-like proteins (6 DEGs) (Table S10).

### Common DEGs identified for comparisons of maintained and abscised flowers, flower pedicels and pods

We identified 28 DEGs that are common for all library comparisons. Almost all of them were regulated in the same direction in the flowers, flower pedicels and pods: 25 DEGs were down-regulated and two up-regulated in all comparisons. The only exception was TIP2:1 (c84944_g1), which was over-expressed in FPNAB and less expressed in FNAP and PNAB (Table 6 and Table S11).

**Table 6:** **List and annotation of common DEGs identified in all three comparison of libraries FAB vs. FNAB, FPAB vs. FPNAB, PAB vs. PNAB**.

**No**	**Unigene ID**	**FAB vs. FNAB**		**FPAB vs. FPNAB**		**PAB vs. PNAB**		**Gene description**
		**log_2_FC**	**Regulated**	**log_2_FC**	**Regulated**	**log_2_FC**	**Regulated**	
1	c107017_g1	−7.17	Down	−4.94	Down	−2.87	Down	tRNA (adenine(34)) deaminase, chloroplastic
2	c107270_g1	−3.82	Down	−3.05	Down	−5.54	Down	No result
3	c107857_g1	−5.65	Down	−2.94	Down	−3.65	Down	Monogalactosyldiacylglycerol synthase 2, chloroplastic
4	c10793_g1	−5.12	Down	−3.23	Down	−3.69	Down	EG45-like domain containing protein
5	c121523_g1	−6.03	Down	−4.83	Down	−4.72	Down	No result
6	c125095_g1	−3.89	Down	−8.00	Down	−3.30	Down	MIR169e
7	c15548_g1	−7.11	Down	−4.21	Down	−3.57	Down	Alpha-aminoadipic semialdehyde synthase
8	c16039_g1	−4.01	Down	−3.19	Down	−2.87	Down	Putative calcium-transporting ATPase 13, plasma membrane-type
9	c27045_g1	−4.12	Down	−2.65	Down	−2.95	Down	Coumaroyl-CoA:anthocyanidin 3-O-glucoside-6″-O-coumaroyltransferase 1
10	c29048_g1	−4.10	Down	−3.72	Down	−2.94	Down	Zinc finger protein ZAT12
11	c3490_g1	−4.81	Down	−5.33	Down	−3.03	Down	Type I inositol 1,4,5-trisphosphate 5-phosphatase 11
12	c3661_g1	−4.34	Down	−7.71	Down	−3.83	Down	Sigma factor binding protein 2, chloroplastic
13	c36657_g1	−4.25	Down	−4.09	Down	−3.09	Down	Heat shock factor protein HSF24
14	c51152_g1	−4.14	Down	−3.89	Down	−3.30	Down	Hydrophobic protein RCI2B
15	c51204_g1	−3.99	Down	−2.98	Down	−3.11	Down	Heat shock factor protein HSF24
16	c51606_g1	−4.40	Down	−4.40	Down	−2.85	Down	Protein QUIRKY
17	c52215_g2	5.36	Up	3.52	Up	3.65	Up	14 kDa proline-rich protein DC2.15
18	c53596_g1	−6.50	Down	−4.06	Down	−2.73	Down	SPX domain-containing protein 3
19	c55641_g3	4.26	Up	3.54	Up	4.16	Up	Endoglucanase 11
20	c56313_g2	−4.32	Down	−3.69	Down	−3.46	Down	No result
21	c57393_g3	−3.91	Down	−5.54	Down	−2.85	Down	Myb-related protein Myb4
22	c58087_g1	−5.11	Down	−4.55	Down	−4.24	Down	Ubiquitin fusion degradaton protein
23	c58367_g2	−4.57	Down	−6.39	Down	−2.98	Down	Beta-glucosidase 12
24	c65572_g1	−4.00	Down	−5.71	Down	−3.08	down	No result
25	c82312_g1	−4.64	Down	−4.02	Down	−3.43	Down	No result
26	c84944_g1	−4.22	Down	2.50	Up	−2.64	Down	Aquaporin TIP2-1
27	c86078_g1	−6.68	Down	−2.59	Down	−3.32	Down	Inorganic pyrophosphatase 2
28	c94313_g1	−5.36	Down	−4.79	Down	−2.73	Down	No result

Among the common DEGs, there were representatives of factors involved in cell wall reorganization (*ENDOGLUCANASE 11, EGL11*), water transport (*TIP2;1*), stress response (for exp. *ZAT12, HSF24, RCI2B, INPP5A, SPX, MYB4*, or *SIB2*) and calcium signaling (*ACA13*). One from common DEGs showing similarity to *QUIRKY* (*QKY*) encoding a predicted membrane-bound protein.

**Figure 5:**
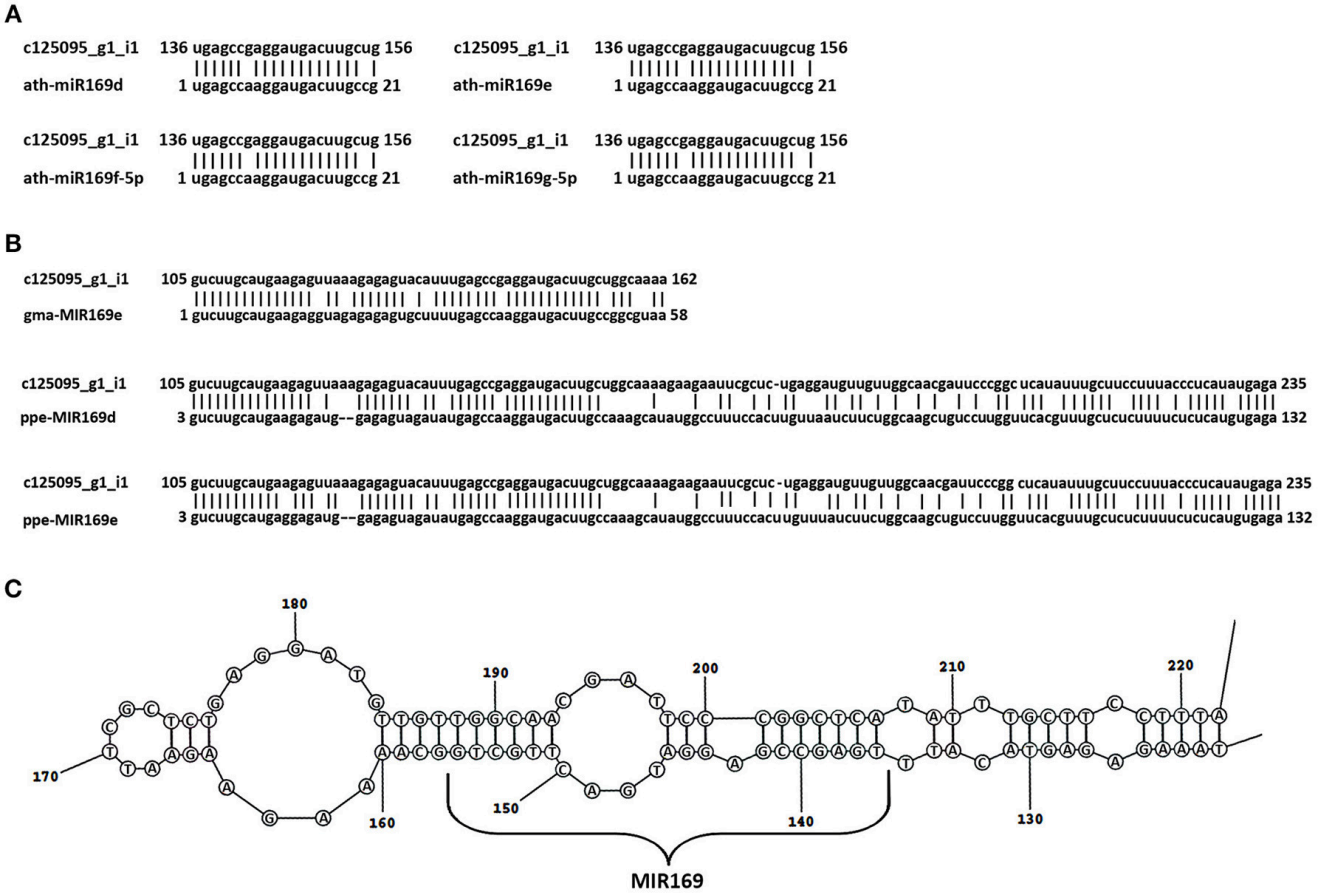
**Identification of the *Lupinus luteus* miR169e precursor**. Alignment of the nucleotide sequence of the c125095g1_i1 unigene with mature miR169d, -e, -f, and -g from *A*. *thaliana* **(A)** and the miR169 precursors from other plant species shows the highest homology **(B)**. miRBase Accession Nos from top to bottom: MIMAT0000908, MIMAT0000909, MIMAT0000910, MIMAT0000911, MI0016576, MI0026086, MI0021629. **(C)** Secondary stem-loop structures of the pre-miR169e of yellow lupine. Localization of mature miR169 is shown on the stem of the precursor sequence.

Among the common DEGs, seven unigenes showed no homology to the protein sequence. A further analysis of these sequences revealed that the c125095_g1 DEG showed similarity to pre-miR169e from *Glycine max* (gma-MIR169e) (Figure 5). This unigene was down-regulated in all cases (Table 6 and Table S11).

### RT-qPCR validation of RNA-seq analysis

In order to confirm the differential expression profiles of the DEGs identified by the RNA-Seq analysis we selected 11 candidates from the set of DEGs common for three library pairs. These were 2 up-regulated and 8 down-regulated DEGs. The RT-qPCR-derived expression patterns of these DEGs fitted well with these determined by the RNA-Seq analysis (Figure S1).

### The enriched pathways (KEGG) analysis of DEGs from compared generative organ libraries

In order to categorize the biological functions of the identified DEGs, we performed a KEGG pathway enrichment analysis (Kanehisa and Goto, 2000; Kanehisa et al., 2016).

### KEGG pathway analysis of DEGs from FAB vs. FNAB comparison

In the flowers, 416 (including 353 up- and 63 down-regulated) DEGs were mapped to 40 KEGG pathways (*p*-value < 0.05) (Table S12). Most of the pathways (32 KEGGs) were part of the major category of “metabolism,” while the others were classified as “genetic information processing” (6 KEGGs), “environmental information processing” (1 KEGG) or “cellular processes” (1 KEGG) (Figure S2A).

**Figure 6:**
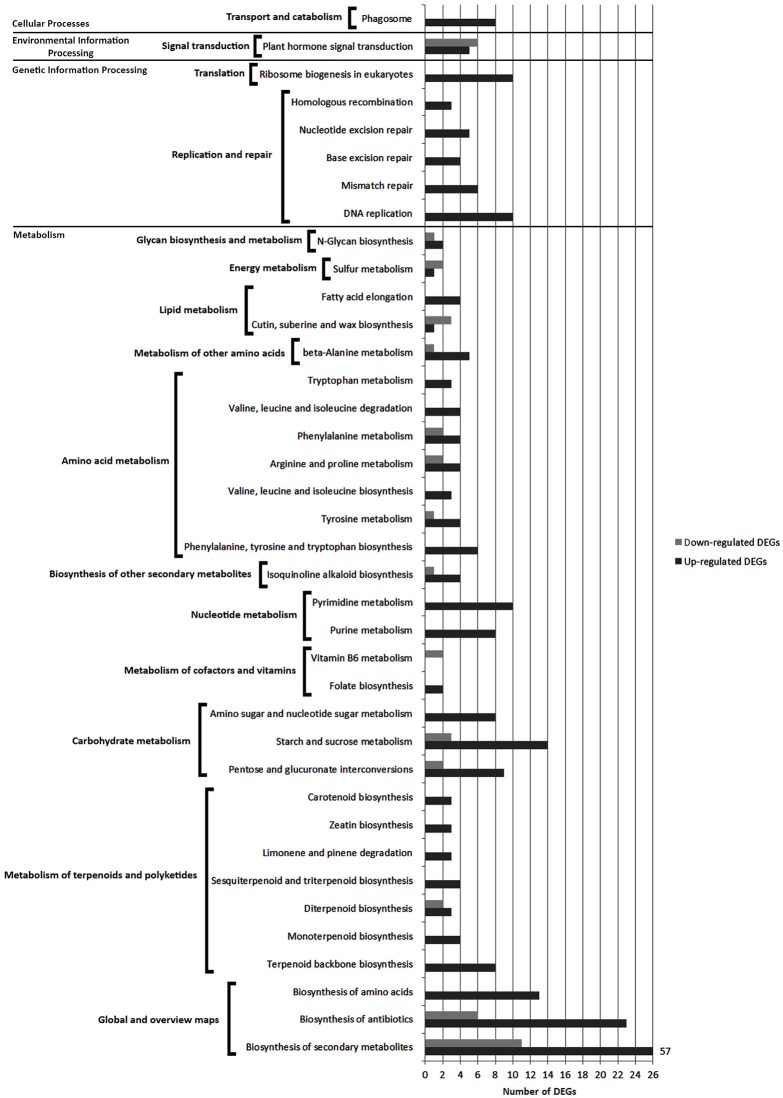
**Comparison of the number of up- and down-regulated DEGs identified in FAB vs. FNAB library comparison in the KEGG pathways**.

Pathways with most numerous DEGs were mapped to the “global/overview maps” category that included “metabolic pathways” [gmx01100] (98 DEGs), “biosynthesis of secondary metabolites” [gmx01110] (68 DEGs) and “biosynthesis of antibiotics” groups [gmx01130] (29 DEGs) (Figure 6 and Figure S2A).

Within the “metabolism” category, 10 general pathways were distinguished, among which the most enriched ones were classified as “carbohydrate metabolism” [gmx00040, gmx00500, gmx00520] (36 DEGs),” “amino acid metabolism” [gmx00400, gmx00350, gmx00290, gmx00360, gmx00330, gmx00280, gmx00380] (33 DEGs) and “metabolism of terpenoids and polyketides” [gmx00900, gmx00902, gmx00904, gmx00909, gmx00903, gmx00908, gmx00906] (30 DEGs) (Figure 6 and Figure S2B).

### KEGG pathway analysis of DEGs from FPAB vs. FPNAB comparison

In the flower pedicels, 715 (including 277 up- and 438 down-regulated) DEGs were mapped to 63 KEGG pathways (*p*-value < 0.05) (Table S13). Most of the pathways (52 KEGGs) were part of the “metabolism” network, and the other ones are classified as “environmental information processing” (3 KEGGs), “cellular processes” (2 KEGGs), “genetic information processing” (1 KEGG) and “organismal systems” (1 KEGG) (Figure S3A).

**Figure 7:**
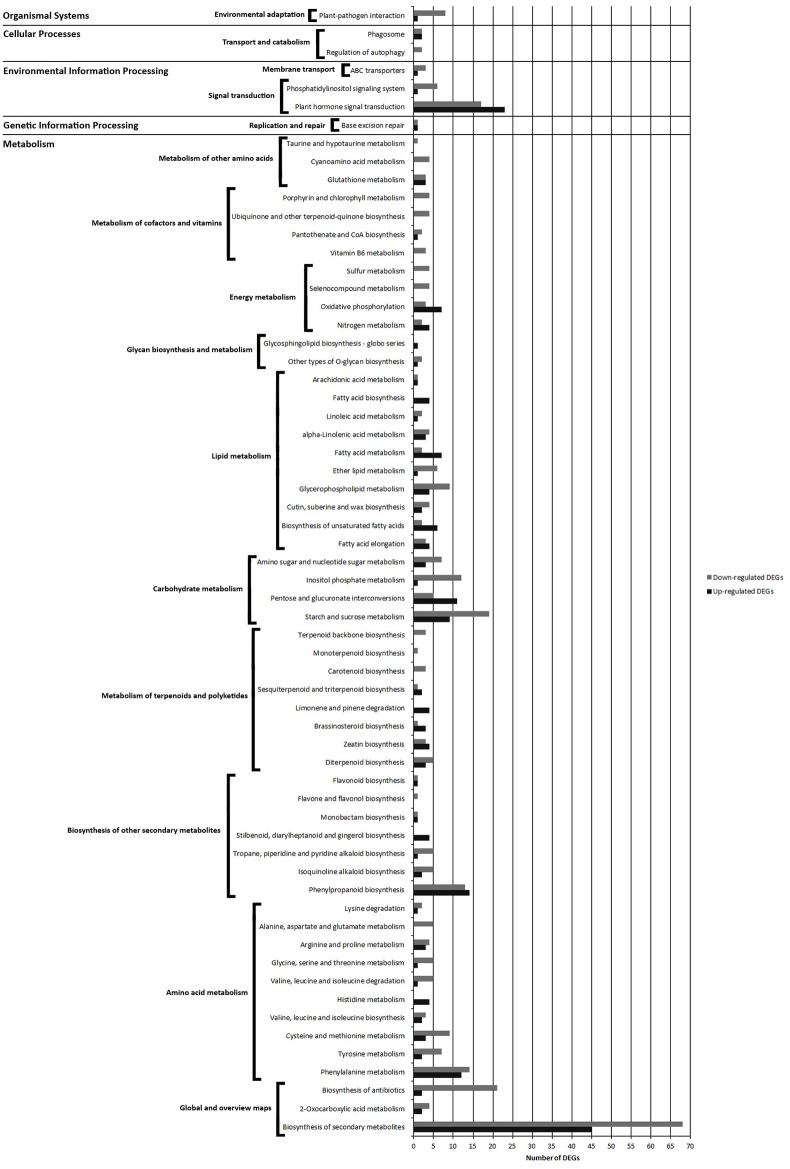
**Comparison of the number of up- and down-regulated DEGs identified in FPAB vs. FPNAB library comparison in the KEGG pathways**.

Pathways with most numerous DEGs were mapped to “metabolic pathways” [gmx01100] (154 DEGs) and “biosynthesis of secondary metabolites” [gmx01130] (113 DEGs) from the “global/overview maps” category, and “plant hormone signal transduction” [gmx04075] (40 DEGs) from the “environmental information processing” category (Figure 7 and Figure S3A).

Within the “metabolism” category, we distinguished 9 general pathways, among which the most enriched ones were classified as “amino acid metabolism” [gmx00360, gmx00350, gmx00270, gmx00290, gmx00340, gmx00280, gmx00260, gmx00330, gmx00250, gmx00310] (83 DEGs), “carbohydrate metabolism” [gmx00500, gmx00040, gmx00562, gmx00520] (67 DEGs) and “lipid metabolism” [gmx00062, gmx01040, gmx00073, gmx00564, gmx00565, gmx01212, gmx00592, gmx00591, gmx00061, gmx00590] (66 DEGs) (Figure 7, Figure S3B).

### KEGG pathway analysis of DEGs from PAB vs. PNAB comparison

In the pods, 508 (including 114 up- and 394 down-regulated) DEGs were mapped to 30 KEGG pathways (*p*-value < 0.05) (Table S14). Most of the pathways (22 KEGGs) were part of the “metabolism” network, and the other ones were classified as “genetic information processing” (2 KEGGs), “environmental information processing” (1 KEGG) and “organismal system” (1 KEGG) (Figure S4A).

**Figure 8:**
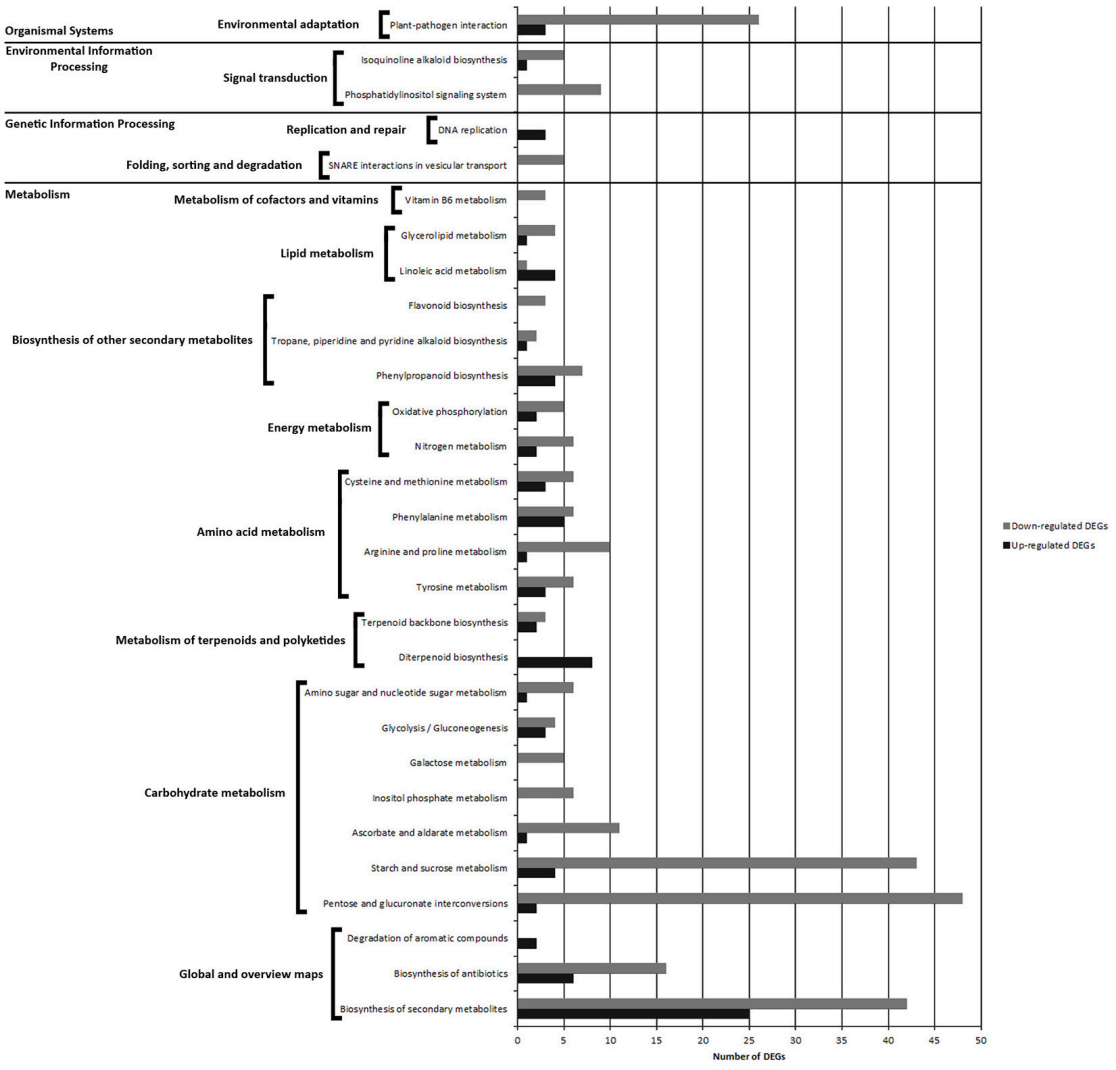
**Comparison of the number of up- and down-regulated DEGs identified in PAB vs. PNAB library comparison in the KEGG pathways**.

Pathways with most numerous DEGs were mapped to “metabolic pathways” [gmx01100] (133 DEGs) and “biosynthesis of secondary metabolites” [gmx01130] (67 DEGs) from the “global/overview maps” category, and “pentose and glucuronate interconversions” [gmx00040] (50 DEGs) from the “metabolism” category (Figure 8, Figure S4A).

Within the “metabolism” category, we distinguished 7 general pathways, among which the most enriched ones were classified as “carbohydrate metabolism” [gmx00040, gmx00500, gmx00053, gmx006562, gmx00052, gmx00010, gmx00520] (134 DEGs), “amino acid metabolism” [gmx00350, gmx00330, gmx00360, gmx00270] (40 DEGs) and “biosynthesis of other secondary metabolites” [gmx00950, gmx00970, gmx00960, gmx00940] (23 DEGs) (Figure 8, Figure S4B).

## Discussion

The process of flower abscission in yellow lupine was investigated already in late 1950s. Those studies demonstrated a significant influence of the flower's location in the inflorescence (Van Steveninck, 1959), the application of various substances, i.e., IAA and TIBA (2,3,5-triiodobenzoic acid, IAA transport inhibitor), and defoliation (Van Steveninck, 1958, 1959) on the degree of flower abortion. Lately, this issue has been revived since it became possible to study flower abscission in *L. luteus* at the molecular level, and several papers have been published (Frankowski et al., 2015; Wilmowicz et al., 2016).

This study represents the first deep-sequencing analysis performed in yellow lupine and focused on flower and fruit abscission. Our analyses allow us to investigate not only genes involved in the molecular mechanism of abscission (in pedicels), but also to compare organs that do and do not drop off the plant, which provides an opportunity to describe the probable causes of abscission. According study conducted on other plant species (Ascough et al., 2005; Corbacho et al., 2013; Estornell et al., 2013; Kim et al., 2015; Sundaresan et al., 2016) we analyzed obtained DEGs with special attention paid to the genes related to hormone and cell wall functioning, and we performed metabolic pathway analyses.

### Transcriptomic changes related to flower and pod abscission in yellow lupine

#### Cell wall related DEGs are associated with organ abscission but also with their development

A number of genes regulate the functioning of the plant cell wall. Changes in their expression are associated with aging (Vetten and Huber, 1990; Han et al., 2016), organ abscission (Kim et al., 2015; Roongsattham et al., 2016), development and maturation of fruits (Brummell et al., 2004; Han et al., 2016; Song et al., 2016; Giné-Bordonaba et al., 2017), and organ growth and development (Gunawardena et al., 2007).

Our RNA-Seq analyses of *L. luteus* generative organs showed bidirectional changes in the expression of the cell wall-related genes, probably associated not only with the ongoing process of abscission but also with progressive development of the organs that are not dropped (Table 2). In a flower transcriptome comparison, we identified 27 DEGs involved in cell wall function. Most of them were up-regulated in FNAB, and only 5 were down-regulated. This is probably related to the inhibition and continuation of growth in flowers being dropped and those remaining on the plant, respectively. This supposition is supported by two facts: (1) non-abscision lupine flowers at this stage of development showed the first signs of ovary growth (data not shown) and (2) pollination in tomato developing flower caused increase in the transcript levels of cell expansion-related genes like *SlEXPA5, SlPEC*, and *SlXTH1* (Vriezen et al., 2007; de Jong et al., 2011). Our results confirm our hypothesis that the abscising flowers are not fertilized, probably because they stopped developing properly even before pollination. In contrast, FNAB at this stage of development showed the first signs of ovary growth that required up-regulation of some cell wall modification-related genes. We strongly believe, that the lack of fertilization is caused by cues triggered from mature flowers on the first whorl and axillary flowers, that elicit modification of expression of specific genes during development of immature flowers. Verification of this hypothesis and identification of these cues need further study.

In our study, the samples were collected at the step of abscission activation (Kim, 2014) (visible lignin accumulation in AZ, Figures 2G,K), where biochemical changes in the cell walls within the AZ are crucial. Many studies (e.g., del Campillo et al., 1988; Lashbrook and Cai, 2008; Roongsattham et al., 2012; Estornell et al., 2013; Tsuchiya et al., 2015) indicate the importance of *PG, CEL, XTH*, and *EXP* genes in organ abscission. Our RNA-Seq analyses showed that in flower pedicels many genes that encoded enzymes responsible for cell wall and middle lamella degradation and remodeling factors, which are the main targets at the late abscission stages, were differently expressed. These included *EXP, XTH, EDG*/*CEL, PG, BGal, PME, PAE, PEC* genes (Table 2 and Table S4), which were previously demonstrated to be highly expressed in the AZs of a large number of abscising organs (Lashbrook et al., 1994; del Campillo and Bennett, 1996; Cho and Cosgrove, 2000; Taylor and Whitelaw, 2001; Roberts et al., 2002; Ogawa et al., 2009; Meir et al., 2010). However, Tucker et al. (2007) discovered that in the soybean leaf AZ most of the cell wall-related genes displayed increased expression, while some of them showed no change or were down-regulated (e g., *EXP1, PG6, PG7, PG16*, and *CEL11*). The authors suggested that different gene products may play very specialized roles in cell wall modifications and down-regulation of some of them may be necessary for the optimal functioning of the others (Tucker et al., 2007). In our case, the identified DEGs showed diverse expression patterns (they were either up- or down-regulated), which may be also related to the fact that we constructed the libraries by taking whole flower pedicels containing not only AZ cells, but also a large number of remaining stalk cells. Additionally, we compared pedicels of growing flowers. The identified DEGs may also be associated with continuation of flower development, which involves the strengthening of pedicels, a further development of vascular bundles, etc. However, active AZ-specific genes (Estornell et al., 2013), namely *CEL1* (c33456_g1), *QRT2* (c56936_g4), *XTH2* (c48301_g1), and *BGAL1* (c59734_g1), were highly expressed in FPAB (Table S4) which indicate that in *L. luteus* organ abscission process are involved similar cell wall modification enzymes as in other plant species.

Pods are shed at the early stages of development (at a length of approximately 1 cm) (Figure 2) (Van Steveninck, 1958), and their location in the inflorescence does not inevitably determine their fate. Pods fall off from higher whorls, but some also from the lowest ones, where most of the pods remain on the plant. They probably compete in some way—the first pod to set has the best chance of achieving full development, while the last one to do so is dropped. Presumably, the signal from older pods causes developmental arrest of younger ones and induce organ abscission. A similar regulation mechanism of shedding fruits is suggested in apple (Botton et al., 2011). In pods of lupine studied here, 76 DEGs were attributed to cell wall metabolism and most of them were over-expressed in the abscising pods that indicate extensive degradation of cell wall structure in these organs (Table 2 and Table S4).

#### Hormonal metabolism and signaling—related genes involved in lupine organ abscission

Plant hormones are involved in regulating all developmental processes in plants (Arteca, 1996) and a large body of evidence indicates that hormones are the critical regulators of organ abscission (for a review see: Kim, 2014; Sawicki et al., 2015). Our analyses of DEGs showed that in yellow lupine genes involved in the auxin and gibberellins metabolism and action presented greatest expression changes, followed by changes in the expression of genes related to the functioning of ABA and ethylene. There were also differences in JA, CK, BR, SA, and STK metabolism.

#### Organs abscission in yellow lupine is associated with intensive changing of auxin catabolism and signaling

Our RNA-Seq analyses indicate that the process of generative organ abscission in yellow lupine is associated with significant changes of auxin balance at the level of catabolism, perception, transport, and response to the phytohormone. The expression of genes encoding auxin biosynthetic enzymes does not differ between the generative organs of *L. luteus* that are shed and those that are not (except for one unigene in flowers). Closer look at the DEGs designed as involved in regulation of auxin availability (metabolism and conjugation) and transport suggest, that during organ abscission there is a huge rebuilding of auxin patterning. In falling flowers this is achieved by inhibition of synthesis, but also break-down and transport of this hormone. In pedicels of falling flowers the pattern probably changes by decreased auxin catabolism and changed transport. In falling pods auxin is probably less available because it is conjugated with proteins. Or it is rather released from conjugates in non-abscission pods.

In the flowers, we identified 11 DEGs involved in auxin signaling (Table 3 and Table S5). Flowers that remain on the plant over-express gene encoding key enzyme in IAA biosynthesisYUCCA8 and DAO, essential for auxin catabolism and maintenance of auxin homeostasis in reproductive organs (Zhao et al., 2013; Panoli et al., 2015). Analyses of auxin biosynthesis mutants (Cheng et al., 2006) indicate that localized IAA synthesis is critical for proper gynoecium morphogenesis in *A. thaliana* (for a review see: Hawkins and Liu, 2014). Up-regulation of *YUCCA8* in FNAP suggests that IAA biosynthesis is also required for proper flower development in yellow lupine. Pollen grains of the *Arabidopsis dao* mutant did not germinate neither *in vitro* nor *in vivo* on the wild-type stigma, and no seeds were produced, which indicates that *DAO* plays a role in regulating anther dehiscence and pollen grain development (Zhao et al., 2013). This suggests that in falling lupine flowers, where *DAO* in less expressed, pollen grains germinate less effectively and in consequence fertilization does not occur, which is the cause of flower abscission. The gene encoding basipetal auxin transporter TORNADO 2 (TRN2) was up-regulated in FNAB. In *Arabidopsis* the auxin pattern resulting from TRN2 activity enables growth and organ organization by cell differentiation (Cnops et al., 2000; Cnops, 2006). Our finding indicate that flower abscission is correlated with inhibition of IAA biosynthesis in one area and simultaneous inhibition of IAA break-down in the other area, and suppression of its transport. This may be caused by cues sent by older flowers, but further study is needed to verify this hypothesis and find possible countermeasures, to increase the number of maintained flowers on the plant. Among the many DEGs analyzed in the flower transcriptomes comparison, two DEGs interpreted as related to IAA signal transduction are worth mentioning: *ARF4* and *ARF7*, up and down-regulated in FNAP, respectively (Table S5). An analysis of tomato *SlARF7* indicate that transcript level of this gene decreased after pollination and transgenic plants with decreased *SlARF7* expression formed parthenocarpic fruits (de Jong et al., 2009). In our RNA-Seq analysis, the *ARF7* mRNA level was higher in the abscising flowers, thus supporting our hypothesis that abscising flowers of *L. luteus* are unfertilizated. DEGs presenting similarity to *ARF4* are present in all comparisons. In the flowers, *ARF4* is over-expressed in FNAB. There is no clear evidence that ARF4 is involved in organ abscission. As genetic analyses indicate, *ARF4* have functions in leaf and floral organ patterning and specify abaxial cell identity (Pekker et al., 2005). In tomato, *SlARF4* expression is high in flowers and young fruits, and decreases during fruit maturation and ripening (Sagar et al., 2013a,b). We suggest that *ARF4* in yellow lupine could be associated with auxin regulation of development by way of symmetric cell division and organ polarity control.

Ethylene and auxin (IAA) are important regulators of abscission and the balance between these phytohormones determines where and when separation takes place (Ascough et al., 2005; Estornell et al., 2013). Most of the unigenes associated with auxin signaling found in this study were identified in the pedicels of flowers (44 out of 70) (Table 4 and Table S6). Two of these DEGs are responsible for auxin break-down: *DAO*, essential for IAA oxidation, and *IAMT1*, involved in formation of MeIAA. MeIAA demonstrates a different activity, as it more easily forms conjugates with proteins and carbohydrates and as a nonpolar molecule probably diffuses through membranes (Qin et al., 2005). Both DEGs were up-regulated in FPNAB. Three of the four DEGs encoding auxin transport proteins were up-regulated in FPNAB, which presumably resulted in a change in auxin distribution. *LAX4* and *LAX5*, encoding the auxin influx carrier (Bainbridge et al., 2008; Petrášek and Friml, 2009; Vanneste and Friml, 2009) proteins were over-expressed in lupine FPNAB. Auxin efflux transporter BIG that controls elongation of pedicels and stem internodes through auxin action in *Arabidopsis* (Gil et al., 2001; Yamaguchi et al., 2007) showed a contrary tendency. Also, the expression of the gene encoding PINOID, a kinase that phosphorylates PINs thus leading to their internalization into cells (Petrášek and Friml, 2009), was reduced. We conclude that in FPAB: (i) IAA break-down was reduced, (ii) the amount of its form that could diffuse out of cells was lower, (iii) auxin influx was enhanced and efflux was inhibited, (iv) and polar auxin transport was reduced. This is consistent with literature data that in poplar leaves prior to cell separation during abscission, an auxin maximum in AZ is formed (Jin et al., 2015) and that reduction of polar auxin transport increases sensitivity of the tissue to ET, which stimulates production of hydrolytic enzymes resulting in organ detachment (Estornell et al., 2013). This indicates, that in yellow lupine the mechanism of organ abscission is similar to that of other plants.

The list of DEGs identified in the pedicels was rich in genes encoding other elements of auxin signal transduction: two encoded down- and up-regulated receptors (F-box protein SKP2A and auxin-binding protein ABP19a, respectively), three encoded down-regulated auxin response factors (ARF4, ARF7 and ARF19), and as many as 15 encoded up- or down-regulated AUX/IAA proteins (Table 4 and Table S6). In *Arabidopsis*, SKP2A is an F-box protein that binds auxin and connects its signaling with cell division (Jurado et al., 2010). Over-expression of *SKP2A* in pedicels of abscising flowers in yellow lupine is probably associated with the auxin-dependent regulation of cell division in the AZ. ABP receptors are known to regulate many processes. Upon auxin binding, they restrict internalization of PINs by inhibiting clathrin-mediated endocytosis (Robert et al., 2010; Grones et al., 2015), thus they are involved in regulating polar auxin transport (Effendi et al., 2011). They also regulate the expression of genes encoding cell wall remodeling proteins, such as EXP, XTH and pectin (Paque et al., 2014). This suggests that during the growth of the pedicel of a flower remaining on the plant, ABP may be engaged in the regulation of cell growth and PIN-mediated auxin patterning. Among auxin-related DEGs in flower pedicels we also found *ARF4* (one DEG up-, another down-regulated in FPNAB), *ARF7* and *ARF19* (both down-regulated in FPNAB). As mentioned before, *ARF4* is probably involved in regulating polar cell division and cell wall modification. Genetic analysis of *Arabidopsis* mutants suggests that *ARF7* and *ARF19* are involved in abscission of floral organs (Estornell et al., 2013). In NGS data analysis of the abscission zone in tomato flowers and leaves *ARF19* was also over-expressed in the AZ, while in soybean leaf abscission only *ARF8* expression changes were observed (Kim et al., 2016). Our results indicate that *ARF7* and *ARF19* can be associated with flower abscission in lupine, but the mechanism of how these genes regulate abscission needs to be clarified.

In the falling pods we observed reduced amounts of *ILR1-like 2* (IAA-amino acid hydrolase) transcripts responsible for IAA conjugation to amino acids (LeClere et al., 2002) and increased DEGs encoding auxin receptors such as TIR1, ABP19, and SKP2A (Table 5 and Table S7). This indicates an increase in free auxin concentrations and a growth of the sensitivity to IAA in these organs. The amount of transcripts encoding ARF2 and ARF4 in PNAB is higher than in PAB. It is suggested that ARF2 is a general repressor of auxin-regulated cell division (Ellis et al., 2005; Okushima et al., 2005) and plays an important role in growth of the ovule before fertilization that determines the final size of the seed (Schruff et al., 2005). Therefore, ARF4 and ARF2 may participate in the regulation of cell division in the developing pods of yellow lupine.

#### Ethylene

In the flower, we found only three DEGs involved in ethylene (ET) signal transduction, which suggests that in this organ ethylene did not play any primary role in abscission regulation (Table 3 and Table S5).

This was not the case, however, in the flower pedicels, as here 17 of the identified DEGs were involved in ethylene metabolism (Table 4 and Table S6). Three unigenes encoding the ET biosynthesis enzyme ACO1 were down-regulated in FPNAB. This confirms the outcome of study suggesting that in *L. luteus*, flowering abortion evoked by ABA resulted from its stimulatory effect on the expression of two main ET biosynthesis genes *LlACS* and *LlACO*, the ACC content and, consequently, the increase in that phytohormone production (Wilmowicz et al., 2016). As mentioned above, ethylene accumulation causes auxin transport inhibition and organ abscission. The same takes place in yellow lupine pedicels, as an increase in the expression of ET biosynthesis genes correlates with decrease in the expression of genes associated with auxin transport in the pedicels of abscising flowers. Additionally, fourteen genes encoding elements of ET signal transduction were up- or down-regulated in FPAB (Table 4 and Table S6). In RNA-Seq analysis of another plant species many genes related to different steps of the ethylene signaling transduction pathway were expressed in the tomato AZs both after flower removal (FAZ) and leaf deblading (LAZ) (Sundaresan et al., 2016). Also, *EIN3* and the genes from ERF family were over-expressed in the tomato flower and leaf AZs (Sundaresan et al., 2016) and in the FPAB of yellow lupine too.

In the pods, all the DEGs attributed to ethylene biosynthesis (ACS1 and ACS7) and signal transduction (ERF3 and ERF1A) were less expressed in PNAB, which indicated that in the abscising pods ET was accumulated in larger amounts (Table 5 and Table S7). Ethylene plays a major role in regulating the expression of cell wall-associated genes encoding PGs (Sitrit and Bennett, 1998; Hiwasa et al., 2004), EXPs (Rose et al., 1997; Hiwasa et al., 2003) and BGals (Karakurt and Huber, 2004; Nishiyama et al., 2007). Therefore, we suggest that an extensive up-regulation of cell wall-related DEGs in the abscising pods could, to some extent, be the result of an increased ethylene biosynthesis in these organs.

#### Gibberellin biosynthesis is induced in organs maintained on plant

Gibberellins are hormones involved in cell expansion, fruit set and growth (Katsumi and Ishida, 1991; Serrani et al., 2007). In contrast to auxin, GA biosynthesis was mainly altered in the tested samples. Changes also occurred at the level of signal transduction and catabolism, but to a minor extent.

The large number of identified genes related to gibberellin metabolism and signaling indicates the importance of GA in lupine flower fate. By comparing the gene expression between the flowers falling off and control, we found 7 DEGs involved in GA functioning. One of them, *GA2OX1* engaged in GA catabolism, was down-regulated, while the others—responsible for its biosynthesis (*KAO2, GA20OX2, GA20OX1*) and signal transduction (*GAI, GASA12*, and *GASA14*)—were up-regulated in FNAB (Table 3 and Table S5). The mRNA levels of GA biosynthesis genes rise after pollination in tomato (Rebers et al., 1999; Serrani et al., 2007). In addition, the transcript level of *SlGA2ox2* was found to be lower, that results in higher levels of GA in active form (Serrani et al., 2008). In *A. thaliana* and *P. sativum*, auxin probably acts as a signal from successfully fertilized ovules that, in turn, stimulates GA biosynthesis and triggers fruit development (Ozga and Reinecke, 2003; Dorcey et al., 2009). Our RNA-Seq analysis shows that probably a similar scenario occurs in yellow lupine.

Similarly to the flowers, in the pedicels of lupine flowers GA biosynthesis was induced in FPNAB (Table 4 and Table S6). This suggests that in this part of non-abscising flowers the GA signaling pathway was also induced.

Our RNA-Seq analysis shows that GA plays an essential role in retaining the pods on the plant (Table 5 and Table S7). The transcription level of GA biosynthesis genes was already higher in FNAB, and the difference even rose in PNAB. All the 10 DEGs attributed to GA biosynthesis were strongly expressed in PNAB, including *GA20OX2* which was the most up-regulated DEG in the developing pods. At the same time, this up-regulation in PNAB means that in the abscising pods lower GA biosynthesis was ceasing their development. Interestingly, DEGs involved in GA signal transduction (mainly *GAMYB*) were significantly over-expressed in the pods being dropped. GAMYB is known as a regulator of flower induction (Gocal et al., 1999) and α-amylase activation in the aleurone layer of the seeds (Gómez-Cadenas et al., 2001; Kaneko et al., 2002). There is no evidence of their function in fruit abscission or development. Only one study demonstrates that in the seeds of *A. thaliana, GAMYB-like* genes are able to promote—but are not essential for—the progression of PCD in the aleurone layer (Alonso-Peral et al., 2010). GA also regulates exine formation and PCD of tapetal cells and the direct activation of *CYP703A3* by GAMYB is crucial for exine formation (Aya et al., 2009). Probably, GAMYB is involved in PCD induction during lupine pod abscission.

#### Involvement of other hormones in lupine organs abscission

There is still little understanding of the participation of other plant hormones in cutting off generative organs. Our study aims to fill this gap.

Our RNA-Seq analysis revealed that ABA receptor *PYL2* was over-expressed in the abscising flowers and flower pedicels (Tables S5, S6). In the flower pedicels, other DEGs recognized as involved in abscisic acid signaling, such as *SAPK2, SAPK8*, and *PP2CA*, were also down-regulated in FPNAB. Simultaneously, *CYP707A4* engaged in ABA break-down was up-regulated. These data suggested that in the abscising flowers and flower pedicels ABA signaling pathways were being activated. In the pods of *L. luteus*, ABA catabolism gene *CYP707A1* was up-regulated in PNAB, which means that in the abscising pods this gene was less expressed, and ABA accumulation occurred. In apple, before fruits abscission a chronological increase in the level of ABA and ACC also takes place (Gómez-Cadenas et al., 2000). Additionally, *ABI3* involved in ABA signal transduction was more expressed in PNAB. ABI3 has been demonstrated to directly induce the expression of storage protein genes (Ezcurra et al., 1999; Reidt et al., 2000; Kroj et al., 2003; Braybrook et al., 2006; Wang and Perry, 2013).

Among the DEGs in the flowers, we identified unigenes that showed homology to jasmonic acid (JA) and ester methyl jasmonate (JaMe) biosynthesis genes (Table S5). A similar situation took place in the pods. DEGs connected with JA biosynthesis were all up-regulated in PNAB (Table 5 and Table S7), which suggests stimulation of metabolism of these hormones in the pods that were continuing to develop. In contrast, in the pedicels of non-abscising flowers, JA biosynthesis was decreased (Table 4 and Table S6). Mutations in JA biosynthesis genes in *A. thaliana* causes male sterility due to delayed anther development and shortened filaments (reviews in Wasternack, 2007; Browse, 2009). Additionally, it was shown that JA is also required for proper development of tomato embryo (Goetz et al., 2012). This again confirms our hypothesis that flowers undergoing abscission are not fertilized and in falling pods embryo development is stopped.

Cytokinins are involved in the regulation of cell division and expansion (Skoog et al., 1965) and CK effect on abscission is thought to be mediated by ethylene (Sipes and Einset, 1983; Grossmann, 1991; Cin et al., 2007). In the pedicels of non-abscising flowers (Table 4 and Table S6), more expressed were those DEGs that showed similarity to: CK receptor AHK4, two genes encoding cytokinin biosynthesis enzymes LOG1 and LOG3, and two genes *CKX5* and *CKX3* encoding enzymes that catalyze CK oxidation to biologically inactive forms (Galuszka et al., 2007). In the pedicels of flowers undergoing abscission two other forms, namely *CKX1* and *CKX9*, were up-regulated. In *Arabidopsis* roots, CKX proteins show diverse subcellular and tissue-specific localization that suggests specific developmental and physiological functions of each gene (Werner et al., 2003). Opposite changes in the expression of *CKX* genes in the flower pedicels of *L. luteus* may be associated with the fact, that different processes regulated by CK occur in various cell compartments and tissues but more study is need to clarify the role of CK in organ abscission in lupine.

Brassinosteroids are endogenous plant hormones essential for the proper regulation of multiple physiological processes required for proper plant growth and development (Clouse and Sasse, 1998; Krishna, 2003; Sasse, 2003; Clouse, 2011). In FPNAB, 8 DEGs were recognized as involved in BR signaling, which suggests that BRs play an important role in the pedicels of the developing flowers (Table 4 and Table S6). Three DEGs involved in signal transduction (Cyclin-D3) were up-regulated in FPNAB, which indicates more intensive cell division in the growing pedicels. DEGs putatively encoding BR biosynthesis enzymes were up- and others down-regulated. BR biosynthesis can be conducted via many alternative pathways which interweave at many nodes and form a sort of metabolic grid (see KEGG map00905; Shimada et al., 2001). In the flower pedicels, some alternative routes are up-regulated, which probably leads to changes not only in BR concentrations, but also in BR composition. BR promote cell expansion through regulation of expression of the genes involved in cell wall modifications, cellulose biosynthesis, ion and water transport, and cytoskeleton rearrangements (Clouse and Sasse, 1998; Schumacher et al., 1999; Morillon et al., 2001; Vert et al., 2005; Kim and Wang, 2010). These findings correlate with our RNA-Seq analysis results; apart from many up-regulated cell wall-related DEGs, 12 DEGs showing similarity to aquaporins were also up-regulated in FPNAB (Table S6).

In the flower pedicels and pods, we identified five and six DEGs (indicating similarity to *TGA, SABP2*, and *PR1*) interpreted as SA-related (Tables 4, 5, Tables S6, S7), respectively. This situation is common during organ abscission. For example, in tomato, transcription factor *TGA* was expressed in FAZ and LAZ (Sundaresan et al., 2016). The transcription of several genes coding for pathogenesis-related (PR) proteins increased concomitantly with the onset of flower abscission of *Sambucus nigra* (Coupe et al., 1997).

The expression of the *DAD2* gene (another name: *DR14*/*DWARF14*) encoding the receptor of SL is differential in the flowers and pods (Tables 3, 5, Tables S5, S7). DR14 protein could work as an intercellular signaling molecule to fine-tune SL function (Kameoka et al., 2016). Recently, a hypothesis in which SL may indirectly influence seed size has been created. SL mutants show enhanced shoot branching and delayed leaf senescence, so they may have reduced nutrient remobilization resulting in a reduction in seed production. These findings indicate that SLs may affect the grain crop yield through leaf senescence and shoot branching regulation (Yamada and Umehara, 2015). We suggest that the increased transcription of the *DAD2* gene in the non-abscising flowers and pods can be linked to the nutrients mobilization.

### Other factors probably involved in generative organ abscission of yellow lupine

#### Transcription factors involved in generative organ abscission

Some transcription factors (TFs) characteristic for soybean leaf and flower abscission processes, such as NAC, WRKY, and AIL/PLETHORA (PLT) (Kim et al., 2016), were also up-regulated in FPAB of lupine (Table S9). Among the DEGs, there were *AIL/PLT* (3 and 2 unigenes respectively), *NAC* (29) and *WRKY* (12). For example, DEG c60377_g2 with log_2_FC = −8.08, a putative ortholog of *NAC* transcription factor 29, also called *NAP* (*NAC-LIKE, ACTIVATED BY AP3/PI*), is closely associated with the senescence process of *Arabidopsis* rosette leaves and, possibly, in other plant species (Guo and Gan, 2006; Zhang and Gan, 2012). The expression of *ANAC019, ANAC055*, and *ANAC072* was induced by drought, high salinity, and abscisic acid (Tran et al., 2004). *NAC055* and *NAC072* were also over-expressed in the FPAB of lupine (Table S9). These results are consistent with previous studies showing up-regulation of this type of TF genes in the flower AZ of tomato (Sundaresan et al., 2016), and the fruit AZ during abscission of mature melon and olive fruit (Corbacho et al., 2013; Gil-Amado and Gomez-Jimenez, 2013). Another transcription factor, namely MYB108, was up-regulated in FAB, as well. This protein contributes to the regulation of stamen maturation and male fertility in response to jasmonate signaling in *A. thaliana* (Mandaokar and Browse, 2008).

Some TFs were more expressed in organs continuing development. For example, the bHLH TF, MYC4 is up-regulated in PNAB (Table S9).The maximum expression of *MYC2, MYC3*, and *MYC4* in *A. thaliana* coincided with the developmental stages during which seed storage reserves accumulated, suggesting these genes may affect the accumulation of seed storage reserves (Gao et al., 2016). Over-expression of *MYC4* in the developing pods of yellow lupine suggests that storage reserves accumulated in seeds.

#### PCD and ROS are associated with abscission process

Organs that completed the abscission, undergo changes that resemble the canonical plant PCD (van Doorn et al., 2011), among others, the stimulation of nucleases and reactive oxygen species (ROS) (Farage-Barhom et al., 2008; Sakamoto et al., 2008a; Meir et al., 2010; Bar-Dror et al., 2011). In tomato, the overexpression of antiapoptotic proteins and inhibition of a PCD-associated ribonuclease trigger abscission (Lers et al., 2006; Bar-Dror et al., 2011), whereas in pepper, ROS inhibitors prevent abscission suppressing H_2_O_2_ production (Sakamoto et al., 2008b).

Our results confirm these findings. Among the DEGs from all the compared libraries we identified numerous genes encoding proteins directly involved in protection against reactive oxygen species, such as peroxidase, as well as enzymes belonging to the carotenoid biosynthesis pathway, such as CCD4, ZDS, and beta-carotene hydroxylase (Tables S8–S10). Also, 16 DEGs displaying similarity to various nucleases were more expressed in FPAB (Table S9). Additionally, one of the most intensively expressed DEGs in the flower pedicels with an active AZ showed similarity to the vacuolar processing enzyme VPE, an executor of plant PCD (Hatsugai et al., 2004). VPE is a cysteine protease that cleaves a peptide bond at the C-terminal side of asparagine and aspartic acid (Wang et al., 2009) and plays an essential role in the regulation of the lytic system of plants during the processes of defense and development (Hara-Nishimura et al., 2005).

#### Water transport plays an important role especially in the developing organs

Our RNA-Seq analysis revealed that water transport played an important role especially in the developing organs. Aquaporin *TIP2;1* was one of the common DEGs for all our study libraries. Additionally, transcripts of 12 DEGs encoding aquaporins belonging to the tonoplast intrinsic protein (*TIP*) and plasma membrane intrinsic protein (*PIP*) gene families were more accumulated in the pedicels of non-abscising flowers (Table S9).There were also 6 (5 up-regulated) and 5 (4 down-regulated) DEGs presenting similarity to genes encoding aquaporins in the flowers and pods, respectively (Tables S8, S10). TIP1 and TIP2 are preferentially associated with the large lytic vacuoles and vacuoles accumulating vegetative storage proteins, respectively (Gattolin et al., 2009). Moreover, intensive expression of *TIP* and *PIP* genes during cell expansion has been observed in numerous plant species (e.g., O'Brien et al., 2002; Liu et al., 2008; Saito et al., 2015). Up-regulation of aquaporin genes in *L. luteus* FNAB and FPNAB is probably associated with cell expansion. On the other hand, in the pods, beside *TIP2;1*, other DEGs belong to the *NIP* aquaporin family (that are involved in transport of water, but also other molecules; Dean et al., 1999; Liu et al., 2003; Wallace and Roberts, 2005; Takano et al., 2006; Choi and Roberts, 2007; Mitani-Ueno et al., 2011) were over-expressed in PAB (Table S10). This was probably associated with the processes of nutrient reutilization.

#### Calcium signaling pathway plays an important role in yellow lupine pod abscission

Calcium (Ca^2+^) is crucial for numerous biological functions. In addition to its key roles in ensuring cell wall and membrane system structural integrity, it has been shown to act as an intracellular regulator in many aspects of plant growth and development, including stress responses (White and Broadley, 2003; Ranty et al., 2006), cell division and elongation, and fruit growth (Hepler, 2005). Recent RNA-Seq analyses of apple fruit abscission revealed that Ca^2+^ deficiency due to the down-regulation of genes encoding transporters of this cation could be a signal for the degeneration of the lateral apple fruits (Ferrero et al., 2015). In contrast, our study the abscising pods demonstrated over-expression of 26 unigenes associated with calcium transport, homeostasis and response (Table S10). Among them there were: a putative calcium-transporting ATPase, a calcium-dependent protein kinase, a probable calcium-binding protein, a cation/calcium exchanger and a calmodulin-like protein. This suggests that the Ca^2+^ signaling pathway plays an important role in yellow lupine pod abscission, although there is a need for further research in order to clarify which physiological processes these changes are associated with.

#### Fertilization probably did not occur in abscising flowers

In addition to the cell-wall related, *MYB108* and other mentioned genes, there were more unigenes associated with pollination that were differentially expressed between the developing flowers and the abscising ones. In the abscising flowers, we detected more mRNA of *MS5*, a gene essential for male fertility, especially for microspora and pollen grain production (Glover et al., 1998), and involved in regulating cell division after male meiosis I and II to facilitate meiotic exit and transition to G1 (Ross et al., 1997; Bulankova et al., 2010) (Table S8). Another gene, *NFD4*, encoding a protein required for polar nuclear fusion during female gametophyte development and karyogamy during fertilization (Portereiko et al., 2006), was also less expressed in FAB. This confirms the hypothesis that shed flowers are unfertilized.

#### Analysis of DEGs common for all comparisons confirms known data but also reveals MIR169 is involved in generative organ abscission

Our analyses of DEGs common for all comparisons allowed determination of which changes were universally associated with the processes of generative organ abscission. By that means we distinguished 28 unigenes with different expression in all of the libraries compared. 25 of them were down-regulated, 2 were up-regulated in all comparisons, and only one—mainly *TIP2;1*—was up-regulated in the FPNAB, unlike the other samples (Table 6, Table S11).

Among the common DEGs, there were representatives of factors involved in the already discussed issues such as cell wall reorganization (EGL11) and water transport (aquaporin TIP2;1). The EG45-like domain containing protein (PNP-A) also has a systemic role in H_2_O and solute homeostasis (Ludidi et al., 2002). Numerous common DEGs are involved in stress response, among them *ZAT12, HSF24, RCI2B, 5INPP5A, SPX3, MYB4*, or *SIB2*. ZAT12 play a central role in reactive oxygen and abiotic stress signaling in *Arabidopsis* (Davletova et al., 2005). SIB2, *Arabidopsis* sigma factor binding proteins, is an activator of the WRKY33 transcription factor in plant defense (Lai et al., 2011). *RCI2B* and *RCI2A* are two developmentally- and stress-regulated cold-inducible genes of *Arabidopsis* encoding highly conserved hydrophobic proteins expressed during the first stages of seed development and germination, in vascular bundles, pollen, and guard cells (Medina et al., 2001).

The presence of the DEG encoding the putative calcium-transporting ATPase 13 (ACA13), the enzyme catalyzing the hydrolysis of ATP coupled with the translocation of calcium from the cytosol out of the cell or into organelles (Iwano et al., 2014), confirmed that calcium signaling plays an important role in organ abscission. Interestingly, among common DEGs we identified one showing similarity to *QKY* encoding a predicted membrane-bound protein. By analogy to animal proteins with related domain topology, QKY is speculated to be involved in Ca^2+^-dependent signaling and membrane trafficking. QKY interacts with receptor-like kinase STRUBBELIG (SUB) at plasmodesm (PD) to promote tissue morphogenesis, for example integument initiation and outgrowth during ovule development (Fulton et al., 2009; Vaddepalli et al., 2014). Our RNA-Seq findings indicate that cell-cell communication *via* QKY was an important factor determining organ fate.

Surprisingly, none of the common DEGs were associated with hormone metabolism or signaling. This was probably due to the fact that although the significant changes in hormone-related genes occurred in all of the tested samples, they were specific to a particular organ. However, the DEGs from the same cluster but with others gene number (c62886_g2, c62886_g7, c62886_g4) indicating similarity to ARF4 proteins were presented in all comparisons (Tables S8–S10).

Our analysis revealed similarity of the common DEG c125095_g1 to the miR169e precursor of *Glycine max* (gma-MIR169e) (Figure 5). MicroRNAs (miRNA) are small noncoding RNAs that control development, stress responses and hormone signaling or metabolism, in both animals and plants, by post-transcriptional regulation of gene expression (Bartel, 2009; Voinnet, 2009). In plants, 21 nt miRNAs are processed from long stem-loop precursor RNAs. The mature miRNA is incorporated into the RNA induced silencing complex (RISC) (Schott et al., 2012) to guide post-transcriptional gene silencing (PTGS) of complementary mRNA by cleavage and/or inhibition of translation (Brodersen and Voinnet, 2009). A sequence analysis revealed that the transcript of unigene c125095_g1 sequence forms the hairpin characteristic for pre-miRNAs, wherein the stem was the sequence of the mature molecule of miR169e (Figure 5), thus indicating that it may be a source of functional mature miRNA. *MiR169* has been identified in many plant species (Sunkar and Jagadeeswaran, 2008) and their isoforms are involved in regulation various processes during development (Gonzalez-Ibeas et al., 2011) and in response to biotic (Singh et al., 2012) or abiotic stresses (Zhou et al., 2010; Licausi et al., 2011; Zhao et al., 2011). In plants, the main targets of miR169 are genes that encode the subunit A of Nuclear Factor Y (Rhoades et al., 2002). In plants, NF-Y TFs have been linked to development (Lotan et al., 1998; Combier et al., 2006; Wenkel et al., 2006), signaling (Warpeha et al., 2007), stress responses (Nelson et al., 2007; Li et al., 2008; Liu and Howell, 2010; Zhao et al., 2011), carbohydrate metabolism and cell wall modification (Leyva-González et al., 2012). Among yellow lupine DEGs no NF-YA transcripts were found, although this may be related to the fact that miR169 may regulate the expression of its target genes also by inhibiting translation, which cannot be detected using RNA-Seq. Further study is needed to identify the processes regulated by miR169 in yellow lupine.

Several DEGs that we identified, such as *ARF2, ARF4* or *GAMYBA* were also regulated by the regulatory sRNAs - miR390, ta-siARF (Marin et al., 2010) and miR159 (Alonso-Peral et al., 2010), respectively. These findings shed new light on possible regulation of organ abscission by non-coding RNAs.

#### RT-qPCR validation of RNA-seq results

In order to validate the results obtained via RNA-Seq analyses, we assessed the trends in expression of 11 chosen genes which displayed significantly different transcription level in all of the compared transcriptomes, using RT-qPCR. The results of the expression analysis of these genes supported the validity of our RNA-Seq (Figure S1).

#### Metabolic changes associated with the processes of generative organ abscission in lupine

KEGG is a highly integrated database providing information on the biological systems and their relationships at the molecular, cellular and organism levels, particularly via the KEGG pathway maps (Kanehisa et al., 2007). An analysis of KEGG metabolic pathways, which was performed using the identified DEGs, allows for a global analysis of the metabolic changes that occur during abscission, both in pedicels with an AZ and the abscising organs. The identified DEGs were grouped into 4 major pathways from the KEGG pathway database (Figures S2A, S3A, S4A): “metabolism,” “genetic information processing,” “environmental information processing” and “organismal systems.” As “metabolism” appeared to be one of the most significant and highly represented categories in our study we ran an in-depth analysis of it, which is presented in Figures S2B, S3B, S4B.

Our analysis of enriched KEGG pathways from all the comparisons indicates that some metabolic pathways were modulated in all the cases, while some seemed to be characteristic for specific plant organs. “Carbohydrate metabolism” and “amino acid metabolism” were common enriched pathway categories for the DEGs from all the comparisons made. This suggests that these metabolism categories were a major target of changes during the abscission of generative organs in yellow lupine. It is worth mentioning that to the “Pentose and glucuronate interconversions” pathway from the “carbohydrate metabolism” category belong i.e., cell-wall related PME and PAE. This explains the path's strong enrichment in all library comparisons, and especially in the pods (Figures 6–8).

The KEGG pathways which were enriched particularly in FNAB were “Terpenoid backbone biosynthesis” from the “Metabolism of terpenoids and polyketides” category (Figure 6). Terpenoids, also known as isoprenoids, are a large class of natural products consisting of isoprene (C5) units. In higher plants, isoprenoids participate in a wide variety of biological functions. Specific examples include photosynthetic pigments (chlorophylls and carotenoids) or hormones (ABA, GA, CK, and BR). The enrichment of these KEGG pathways in the flower could be associated with hormone biosynthesis. Other groups of up-regulated DEGs in FNAB belonged to the “Genetic Information Processing” category, and particularly to the “Replication and repair” and “Translation” pathways (38 DEGs) (Figure 6 and Figure S2A). The fact that, apart from these pathways, amino acid biosynthesis and metabolism and nucleotide metabolism pathways were enriched, too, indicates that cell division and intensive *de novo* protein synthesis were induced in FNAB or from the other side these processes are inhibited in FAB.

Other pathways from the KEGG metabolism category in which DEGs identified in flower pedicels were indexed fell under the “Environmental Information Processing” heading, and included the “Plant hormone signal transduction pathway,” which confirmed the importance of changes in hormonal balance in the studied pedicels (Figure 7). This could be associated with the “Inositol phosphate metabolism” pathway that was over-represented in FPAB. We found that DEGs showing similarity to, *inter alia*, non-specific phospholipase C (NPC), belonged to this pathway. NPCs play important roles in many processes as phospholipid-to-galactosyl DAG exchange, growth and development associated with hormone signaling and stress responses (reviewed in: Pokotylo et al., 2013). The same phospholipases were included in the “Ether lipid metabolism” and “Glycerophospholipid metabolism” pathways that were strongly up-regulated in the abscising flower pedicels. In contrast, the “Biosynthesis of unsaturated fatty acid” and “Fatty acid metabolism” pathways that are probably associated with cell membrane functioning were enriched in the flowers that continued to develop.

In the pods, the “Plant-pathogen interaction” pathway from the “organismal systems” major category was strongly enriched due to high numbers of up-regulated DEGs related to PR1, calcium-related genes and WRKY in PAB (Figure 8). Another pathway enriched in the abscising pods was “Phosphatidylinositol signaling system,” which include DEGs encoding calmodulines and phospholipase A and C. In PAB, within the “amino acid metabolism” category we found DEGs showing similarity to enzymes involved in protein degradation, such as Cysteine proteinase (e.g., c134689_g1, c73956_g1, Table S10) (Martínez et al., 2012) or Proline dehydrogenase (e.g., c60845_g3, c53844_g2, Table S10) (Funck et al., 2010; Monteoliva et al., 2014), which confirmed our DEGs analysis indicating a strong induction of degradation processes in PAB. Only one pathway, “diterpenoid biosynthesis” related to GA biosynthesis, was clearly enriched in PNAB, which fits well to our analysis of hormone-related DEGs in the pods.

## Conclusions and future perspectives

In our work we analyzed global gene expression in the flowers, flower pedicels, and pods collected from *L. luteus* with the purpose of elucidating the molecular mechanisms and metabolic pathways involved in physiological abscission, and assessing the role of generative organs in this process.

As it might be expected, the process is regulated comprehensively and leads to the modification of gene expression, consequently causing hormonal, metabolic, and structural changes. The expression of certain genes changes in all of the compared libraries, while that of others is modified specifically in particular organs.

**Figure 9:**
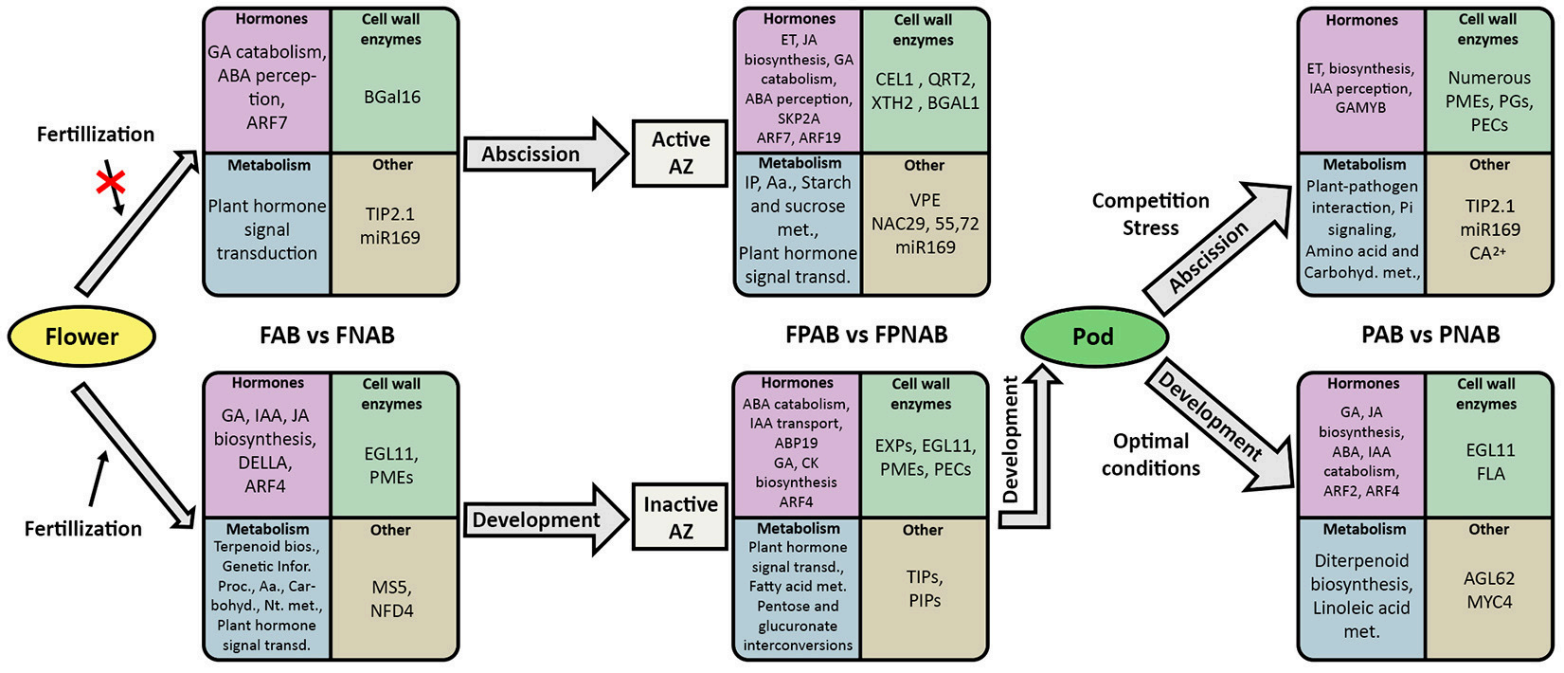
**Diagram showing the complex changes in yellow lupine abscising generative organs and organs continuing their development on the basis of the obtained results**.

We propose a model, in which (Figure 9), unfertilized flowers of *L. luteus* induce the process of falling off and the activation of the AZ in the flower pedicel. In the fertilized flowers, changes related to further development and pod formation occur. The pedicels of these flowers also undergo structural modifications associated with the transport of water and nutrients and the maintenance of the growing fruit. The fate of the formed pods depends upon the sequence of establishing and the environmental conditions. Under unfavorable conditions growth is arrested, nutrient reutilization processes are activated, and cell wall degradation and PCD processes are launched. In all the cases, changes occurred in respect to the expression of a number of genes associated with the functioning of the cell walls, hormones and the metabolism of sugars and amino acids. Also, differences in the metabolism of hormones in the pedicels, as well as the signaling system and stress response in the pods, were observed. The observed increased *MIR169* expression in all of the abscising organs was a new discovery.

This study provides promising advances in our understanding of generative organ abscission that may ultimately lead to improved control of *L. luteus* flower and pod set. In order to establish a more precise model and identify the abscission signal trigger, further studies are necessary. Further research is needed also to resolve whether here described changes in generative organs initiate before or after AZ activation, and whether they are the cause or result of abscission. Nevertheless, our study represents an important step in elucidating the biological pathways engaged in generative organ abscission, and more research is being conducted in order to unravel new genes and functions involved in this process.

## Author contributions

PG conception and design of the experiment, plant and RNA sample preparation, data analysis, interpretation of data and wrote the manuscript; WW, KM, NK, and JKe conception and design of the experiment, plant and RNA sample preparation; WG and MK data analysis and manuscript preparation; JKo conception of the experiment and interpretation of data for the work.

## Funding

This research was funded by the Polish Ministry of Agriculture and Rural Development grant No. 149/2011, the program supported by Resolution of the Council of Ministers (RM-111-222-15) in association with the Institute of Plant Genetics, Polish Academy of Sciences, and by The National Science Centre SONATA grant No. 2015/19/D/NZ9/03601 and NCN grant No. 2011/01/B/NZ9/03819.

### Conflict of interest statement

The authors declare that the research was conducted in the absence of any commercial or financial relationships that could be construed as a potential conflict of interest.

## Supplementary material

Figure S1 **qPCR validation of differential expression patterns of selected unigenes**. The relative quantification of the gene expression level was determined with qPCR assay using *ACTIN* as a reference gene. The values presented on top of each bar indicate the expression level derived from the RNA-Seq data.

Figure S2 **Annotation of *L*. *luteus* DEGs from an FAB vs. FNAB library comparison by KEGG database. (A)** Distribution of DEGs into KEGG biological categories. The numbers of KEGG pathways belonging to a category are shown in brackets. **(B)** Classification of DEGs into the KEGG “Metabolism” category. The numbers of DEGs belonging to a pathway are shown.

Figure S3 **Annotation of *L*. *luteus* DEGs from an FPAB vs. FPNAB library comparison by KEGG database. (A)** Distribution of DEGs into KEGG biological categories. The numbers of KEGG pathways belonging to a category are shown in brackets. **(B)** Classification of DEGs into the KEGG “Metabolism” category. The numbers of DEGs belonging to a pathway are shown.

Figure S4 **Annotation of *L*. *luteus* DEGs from comparison of PAB vs. PNAB libraries by KEGG database. (A)** Distribution of DEGs into KEGG biological categories. The numbers of KEGG pathways belonging to a category are shown in brackets. **(B)** Classification of DEGs into the KEGG “Metabolism” category. The numbers of DEGs belonging to a pathway are shown in brackets.

Table S1 **List of primers used for the RT-qPCR validation and the RT-qPCR validation results**.

Table S2 **Quality filtering and statistics of raw reads obtained for eight transcriptome libraries of *L*. *luteus***.

Table S3 **Statistical analysis of differentially expressed gene in compared libraries of *L. luteus***.

Table S4 **Differential expression patterns of cell wall-related genes in library comparison of FAB vs. FNAB, FPAB vs. FPNAB, PAB vs. PNAB**.

Table S5 **Differential expression patterns of plant hormone signaling-related genes in library comparison of FAB vs. FNAB**.

Table S6 **Differential expression patterns of plant hormone signaling-related genes in library comparison of FPAB vs. FPNAB**.

Table S7 **Differential expression patterns of plant hormone signaling-related genes in library comparison of PAB vs. PNAB**.

Table S8 **List and annotation of differentially expressed genes (DEGs) between FAB and FNAB with PPDE < 0.05**.

Table S9 **List and annotation of differentially expressed genes (DEGs) between FPAB and FPNAB with PPDE < 0.05**.

Table S10 **List and annotation of differentially expressed genes (DEGs) between PAB and PNAB with PPDE < 0.05**.

Table S11 **List and annotation of common DEGs identified in all three library comparisons of FAB vs. FNAB, FPAB vs. FPNAB, PAB vs. PNAB with FPKM (Fragment Per Kilobase of exon per Million fragments mapped) in each of the libraries**.

Table S12 **The enriched pathways (KEGG) of DEGs from library comparison of FAB vs. FNAB**.

Table S13 **The enriched pathways (KEGG) of DEGs from library comparison of FPAB vs. FPNAB**.

Table S14 **The enriched pathways (KEGG) of DEGs from library comparison of PAB vs. PNAB**.
