# Supplementary figures and images for: De novo Transcriptome Profiling of Flowers, Flower Pedicels and Pods of Lupinus luteus (Yellow Lupine) Reveals Complex Expression Changes during Organ Abscission
Source: Front Plant Sci. 2017 May 2;8:641. doi: 10.3389/fpls.2017.00641 (PMC5412092; doi:10.3389/fpls.2017.00641)

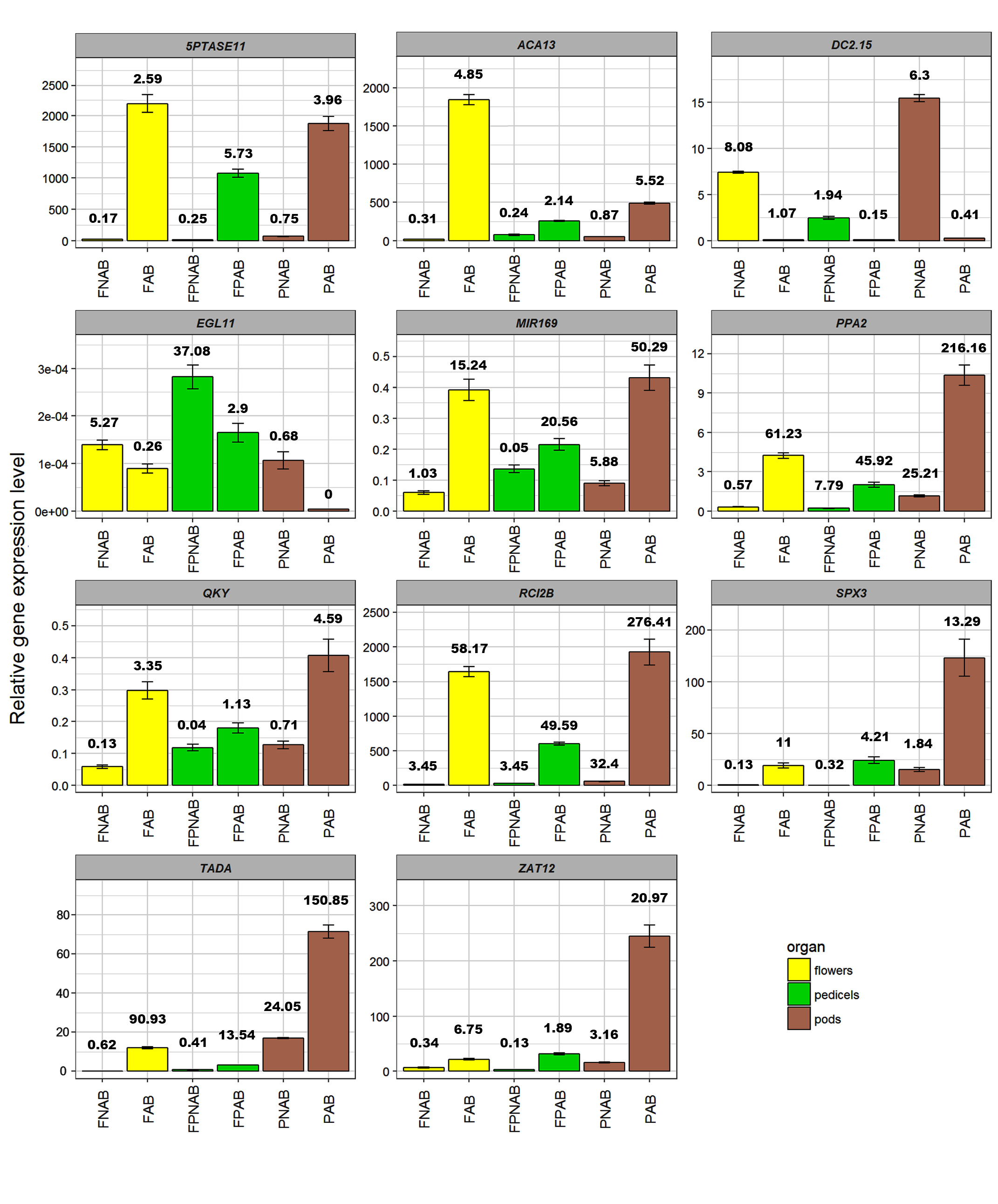

Supplement: Figure S1 — qPCR validation of differential expression patterns of selected unigenes. The relative quantification of the gene expression level was determined with qPCR assay using ACTIN as a reference gene. The values presented on top of each bar indicate the expression level derived from the RNA-Seq data. [file FigureS1.jpeg]

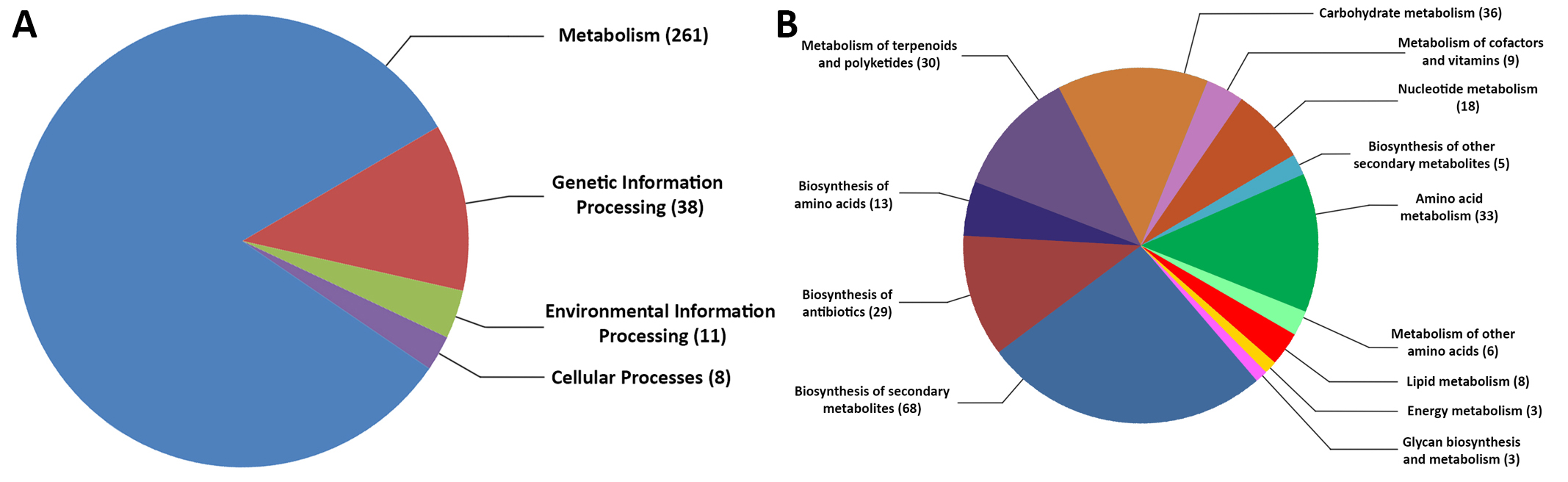

Supplement: Figure S2 — Annotation of L. luteus DEGs from an FAB vs. FNAB library comparison by KEGG database. (A) Distribution of DEGs into KEGG biological categories. The numbers of KEGG pathways belonging to a category are shown in brackets. (B) Classification of DEGs into the KEGG “Metabolism” category. The numbers of DEGs belonging to a pathway are shown. [file FigureS2.jpeg]

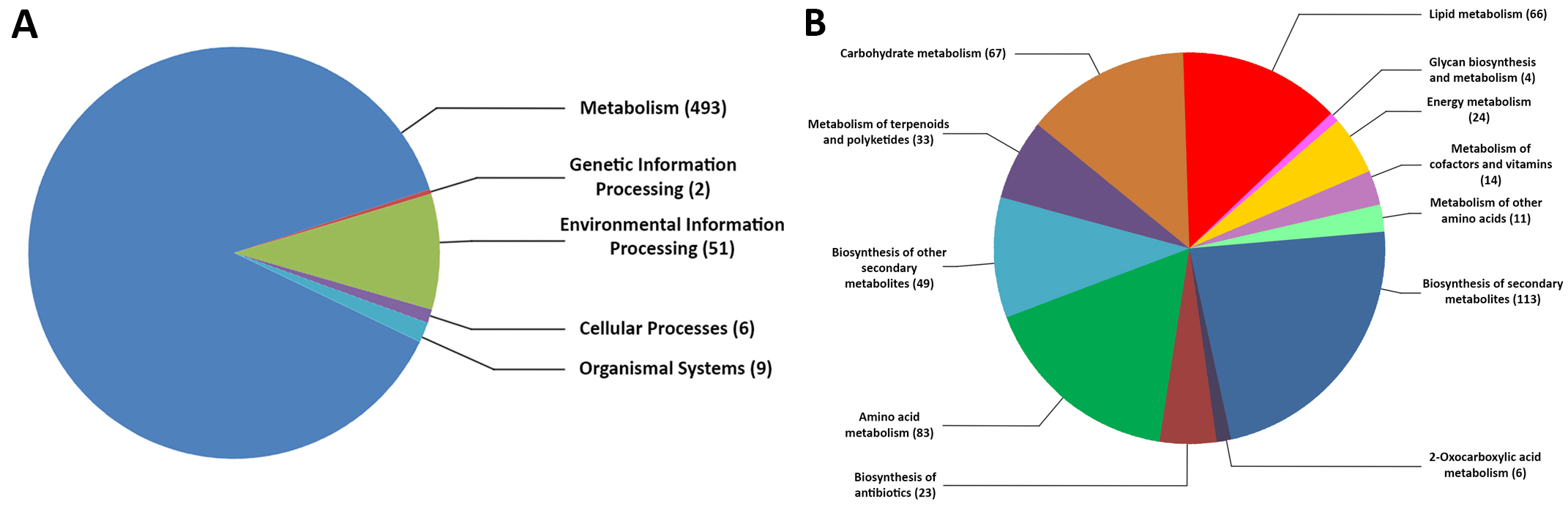

Supplement: Figure S3 — Annotation of L. luteus DEGs from an FPAB vs. FPNAB library comparison by KEGG database. (A) Distribution of DEGs into KEGG biological categories. The numbers of KEGG pathways belonging to a category are shown in brackets. (B) Classification of DEGs into the KEGG “Metabolism” category. The numbers of DEGs belonging to a pathway are shown. [file FigureS3.jpeg]

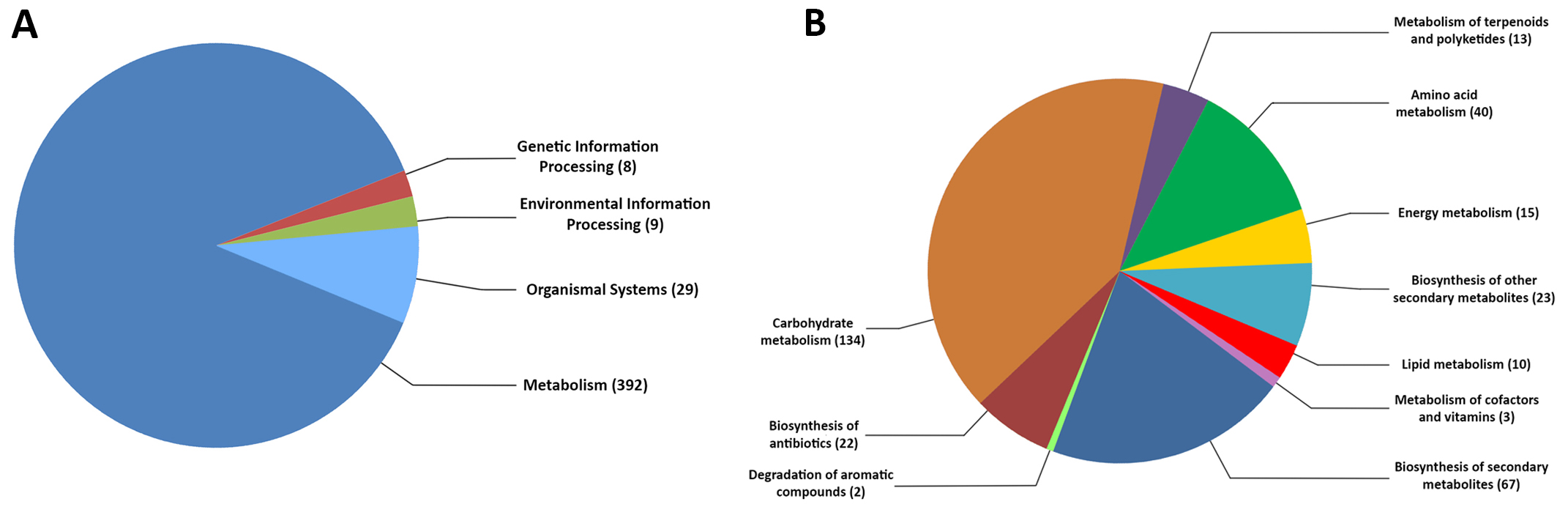

Supplement: Figure S4 — Annotation of L. luteus DEGs from comparison of PAB vs. PNAB libraries by KEGG database. (A) Distribution of DEGs into KEGG biological categories. The numbers of KEGG pathways belonging to a category are shown in brackets. (B) Classification of DEGs into the KEGG “Metabolism” category. The numbers of DEGs belonging to a pathway are shown in brackets. [file FigureS4.jpeg]
